# Warming increases trophic cascade strength in an aquatic food chain

**DOI:** 10.1111/1365-2656.70290

**Published:** 2026-06-02

**Authors:** Francis P. Biagioli, Kyle E. Coblentz, Liuqingqing Yang, Dinelka Thilakarathne, John P. DeLong

**Affiliations:** ^1^ School of Biological Sciences University of Nebraska‐Lincoln Lincoln Nebraska USA; ^2^ Present address: Department of Biology Colby College Waterville Maine USA

**Keywords:** consumer–resource interactions, global climate change, indirect effects, predator–prey interactions, species interactions, stability, top‐down and bottom‐up effects, trait‐mediated effects

## Abstract

1. Trophic cascades play a central role in shaping ecosystems, yet it is unclear how their strength responds to warming. Because species' demographics and trophic interaction strengths are temperature sensitive, climate change is expected to alter cascade strengths, with potentially widespread ecological consequences.
2. We experimentally tested how temperature affects trophic cascade strength by manipulating the presence of the predator *Hydra oligactis* and tracking the abundances of its prey, *Ceriodaphnia reticulata*, and primary producer, *Ankistrodesmus falcatus*, across a temperature gradient.
3. To uncover the mechanisms driving these changes, we complemented the experiment with mathematical models fit to the population dynamics, providing novel insight into *why* trophic cascade strength changes with warming.
4. We predicted that warming would strengthen trophic cascades by increasing direct consumer–resource interaction strengths. Our results supported this prediction, and we also found that higher temperatures amplified transient population fluctuations, driven by the combined temperature dependence of nearly all the model parameters.
5. Our findings show that climate warming can strengthen trophic cascades, destabilize population dynamics and magnify the ecological impacts of predator loss through complex, temperature‐dependent changes in species interactions and demographics.

## Graphical abstract

Using experiments and Bayesian dynamical modelling, we demonstrate that warming strengthens trophic cascades in an aquatic food chain through coordinated, temperature‐dependent shifts in predator, prey and resource traits. By tracing indirect effects to underlying mechanisms, our study shows how climate change can amplify predator impacts and destabilize food webs.

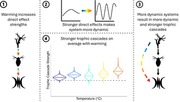

## 1 INTRODUCTION

Trophic cascades, the indirect effects of predators on lower trophic levels, strongly influence ecosystem structure and function (Carpenter & Kitchell, 1988; Li et al., 2023; Paine, 1980; Ripple et al., 2016). They occur in all ecosystems (Knight et al., 2005), involve a wide diversity of taxa (Shurin et al., 2002) and alter community structure by causing alternating decreases and increases in species abundance across trophic levels (Carpenter et al., 2008; Terborgh & Estes, 2014; Wootton, 1994b). These shifts mediate the consequences of top predator extirpation (Estes et al., 2011; Lundgren et al., 2022; Ripple et al., 2025), pest impacts on agricultural yields (Costamagna et al., 2007) and eutrophication (Zhang et al., 2023), while also influencing biogeochemical cycles and greenhouse gas emissions (Atwood et al., 2013; Strickland et al., 2013; Wilmers et al., 2012).

The strength and prevalence of trophic cascades depend on interacting ecological and environmental factors (Borer et al., 2005; Terborgh & Estes, 2014), including predator and prey taxonomy, body size ratios, foraging characteristics, habitat type and size, food web length, system productivity, resource availability and climate (Brett & Goldman, 1996; DeLong et al., 2015; Kratina et al., 2012; Otto et al., 2008; Rodríguez‐Castañeda, 2013; Schmitz et al., 2004; Stevenson et al., 2016; Su et al., 2021). These factors influence trophic interactions and can alter the way trophic cascades propagate through a food web (Berlow et al., 2009).

Trophic cascades emerge from chains of linked direct effects, and their strengths are functions of the direct effect strengths in a food chain (DeLong et al., 2015; Paine, 1980; Ripple et al., 2016; Wootton, 1994b). Direct effects are those of one population on another through a combination of interactions among individuals (e.g. consumption) and population demographics (birth and death rates) that determine species abundances (Wootton, 1994a). Because direct effects link populations, shifts in their relative strengths determine whether and how top‐down effects propagate through a food chain, thereby shaping the magnitude of predator impacts on resource abundances (Menge, 1995; Novak et al., 2016; Schmitz & Suttle, 2001; Wootton, 1994b). For example, changes in consumer conversion efficiency can modify predator birth rates, reducing the effect of predators on prey populations, thereby weakening the regulation of resource abundances and ultimately the strength of trophic cascades (Zhou et al., 2025). Similarly, plant defences can shift herbivore foraging behaviour, changing herbivore–plant direct effect strengths and therefore the strength of trophic cascades (Mooney et al., 2010).

Temperature likely shapes the strength of direct effects in food chains involving ectotherms, as many ectotherm biological processes are temperature‐dependent (Huey & Berrigan, 2001; Jarośík et al., 2004). Laboratory and field studies show that ectotherm predator functional responses (the per capita foraging rate of a predator across prey densities) vary with temperature (Coblentz et al., 2022; Englund et al., 2011; Rall et al., 2012; Uiterwaal & DeLong, 2020; West & Post, 2016). In parallel, demographic processes at the population level (captured by intrinsic growth rates and carrying capacities) generally show non‐linear temperature dependencies, indicating complex effects of temperature on resource dynamics (Bieg & Vasseur, 2024; Mallard et al., 2020; Stockseth et al., 2025).

Given ongoing global climate change, it is critical to understand how temperature alters trophic interactions and cascade strengths. Generally, studies predict stronger trophic cascades with warming (Borer et al., 2005; Kratina et al., 2012; Ripple et al., 2024; Rodríguez‐Castañeda, 2013; Su et al., 2021), but few have tested this prediction directly. To evaluate whether warming strengthens trophic cascades and to quantify underlying mechanisms, we conducted a laboratory experiment tracking the abundances of a daphnid consumer (*Ceriodaphnia reticulata*) and an algal resource (*Ankistrodesmus falcatus*) in the presence and absence of a cnidarian predator (*Hydra oligactis*) across a temperature gradient. We measured trophic cascade strength as the ratio of primary producer abundances with and without the predator, a commonly used and standardized metric in trophic cascade research that facilitates comparisons across systems and treatments (Borer et al., 2005; DeLong et al., 2015; Shurin & Seabloom, 2005). This approach allows trophic cascade strength to be interpreted directly in terms of changes in resource abundance through time, facilitating mechanistic links between temperature‐driven population dynamics and cascade strength. We then compared these strengths across temperature treatments. We chose the *Hydra–Ceriodaphnia–Ankistrodesmus* system because the species have relatively short generation times, are easy to culture, co‐occur widely in nature and share similar temperature tolerance ranges (Bosch et al., 1988; Lürling et al., 2013; Schroeder & Callaghan, 1981).

Because trophic cascades depend on direct effects within food chains, any temperature‐driven change in trophic cascade strength must result from changes in those direct effects generated by both foraging interactions and demographic processes (Antiqueira et al., 2022; DeLong & Lyon, 2020; Gilbert et al., 2014; Menge, 1995; Preisser & Strong, 2004). Yet the biological mechanisms driving stronger trophic cascades remain unclear. In addition, there is considerable nonlinearity in the effects of temperature on foraging (Burnside et al., 2014; Rall et al., 2010; Uiterwaal & DeLong, 2020), reproduction (Rebolledo et al., 2020; Savage et al., 2004) and mortality (McCoy & Gillooly, 2008), making it difficult to predict how these processes interact across trophic levels to influence the overall magnitude of trophic cascades. Here, we address these knowledge gaps by fitting predator–prey models to our experimental time series data, allowing us to understand how specific parameters representing fundamental biological processes change with temperatures and to trace the consequences of warming from direct effects to population dynamics and ultimately the strength of trophic cascades.

We found that average trophic cascade strength increased with temperature, driven by a strong but transient increase in trophic cascade strength across warmer temperatures. This pattern reflected shifts in foraging interactions, increased resource growth at warmer temperatures and temperature‐dependent non‐consumptive effects of predators on consumers. Together these results not only indicate that temperature can alter trophic cascade strengths but also show that these changes occur via a complex suite of ecological mechanisms.

## 2 MATERIALS AND METHODS

### 2.1 Culturing and experimental design

We collected *H. oligactis* (hereafter hydra) from Holmes Lake, Lincoln, NE, USA (40.7816, −96.6343) and *Ceriodaphnia reticulata* (hereafter daphnia) from Spring Creek Prairie Audubon Center, Southwest of Lincoln (40.6928, −96.8519). We acquired *Ankistrodesmus falcatus* (hereafter algae) from the Culture Collection of Alga at University of Texas at Austin (UTEX). We maintained stock cultures of all species at 19°C in filtered, autoclaved pond water (ACPW) from the daphnia source pond. To prepare ACPW, we filtered pond water twice through 185 mm Whatman filter paper to remove organic matter and non‐study organisms (CAT No. 1001‐185, Cytiva, Global Life Sciences Solutions USA LLC, 100 Results Way, Marlborough, MA USA). We then autoclaved the water at 121.1°C for 20 min and filtered it a third time to remove unwanted particulates (Spire Integrated Solutions, 8719 S 135th St #300, Omaha, NE, USA). We grew algae using 5% Alga‐Gro media (Carolina Biological Supply, Burlington, NC, USA) diluted to 5 mL: 1 L Alga‐Gro:ACPW. We fed daphnia with algae cultures and fed hydra a mix of *C. reticulata* and *Daphnia magna* (Kaimes Farm, Leitholm, Scottish Borders).

We assembled fifty 300 mL mesocosms in 473 mL straight‐sided, wide‐mouth glass jars with lids, completing assembly within two hours. Each jar received 20 daphnia and 100 mL of ACPW, followed by 150 mL of algae‐inoculated ACPW. We then filled all jars to 290 mL with ACPW and added two hydra to 25 randomly chosen jars. Finally, we filled all jars to 300 mL with 5% Alga‐Grow media.

We randomly assigned jars to one of five temperatures (14, 17, 20, 23 and 26°C) with five predator and five no‐predator replicates per temperature. We chose this range to ensure hydra survival, since trophic cascades can only be observed in temperatures that both predator and prey can tolerate (Bosch et al., 1988; Schroeder & Callaghan, 1981). We maintained mesocosms in Percival incubators (Model #: E30B; Percival Scientific, Inc. 505 Research Drive Perry, IA, USA) under a 12h:12 h light:dark cycle at 65% humidity. We split replicates evenly across treatments into two groups started on consecutive days.

We counted hydra, daphnia and algae in each replicate every other day for 35 days. We censused hydra visually by scanning each jar over a light table. To count daphnia, we used a scaled sampling technique approach: We visually censused the full volume of each jar for daphnia at low densities (<100 daphnids total) and we removed and counted a 10‐mL sample over a light table at high densities (>100 daphnids). In addition, we removed 10 mL from visually censused jars to maintain consistency between jars counted with differing sampling techniques. We estimated algae densities from a 10 μL sample using a Neubauer haemocytometer (Model # 1280; Electron Microscopy Sciences. 1560 Industry Road, Hatfield, PA, USA) under an AMG EVOS xl core inverted microscope (Model # AMEX‐1000; Advanced Microscopy Group, Bothell WA, USA). We randomly selected ten 6.25 × 10^−6^ mL subsamples and summed these counts to calculate algae abundance in 6.25 × 10^−5^ mL. To maintain semi‐continuous batch cultures, we replaced the 10 mL removed during daphnia censusing with fresh 5% Alga‐Gro media. We excluded one predator‐absent replicate at 20°C treatment from our analysis because algae populations failed to establish. All mesocosms included in the analyses retained all species throughout the experiment; no extinctions occurred beyond the excluded replicate in which algae failed to establish. Our study did not require ethical approval from an animal ethics committee.

### 2.2 Dynamical population model

We fit ordinary differential equation (ODE) models to time series data to estimate parameters, generate population dynamics solutions and calculate trophic cascade strengths. We used the following ODE model to describe the interacting populations of daphnia (*C*, individuals/mL) and algae (*R*, individuals/mL) in the absence of hydra:

(1A) $$\frac{\mathrm{dR}}{\mathrm{dt}}=\mathrm{rR}\left(1-R*{\mathrm{Qe}}^{\mathrm{Bt}}\right)-\frac{a_{c}\mathrm{RC}}{1+a_{c}h_{c}R+w_{c}\left(C-0.0033\right)}$$

(1B) $$\frac{\mathrm{dC}}{\mathrm{dt}}=\frac{e_{c}a_{c}\mathrm{RC}}{1+a_{c}h_{c}R+w_{c}\left(C-0.0033\right)}-d_{c}C$$

In this model, *r* is the intrinsic rate of population growth for algae [days^−1^], and *Q* is the per capita reduction in growth rate due to intraspecific competition (note that *Q* = 1/carrying capacity). The two equations are linked by a Beddington–DeAngelis functional response (Beddington, 1975; DeAngelis et al., 1975; DeLong & Vasseur, 2011; Holling, 1959) where *a*_*c*_ is the daphnia space clearance rate (i.e. the volume of habitat cleared of resources per consumer per time in the absence of a time cost to handling constraints [mL consumer^−1^ days^−1^]) on algae, *h*_*c*_ is the handling time (i.e. time cost to further searching from capturing and consuming resources [days resource^−1^]), *w*_*c*_ is interference among daphnia (i.e. search area missed per consumer [area or volume missed consumer^−1^]), *e*_*c*_ is the efficiency of converting algae into new daphnia [consumers per resource], *d*_*c*_ is the daphnia background death rate [days^−1^] and *t* is time [days]. We included a time‐dependent term *B* that modifies *Q* to capture declining environmental quality over the course of the experiment, which was necessary to achieve model fits, especially at warmer temperatures. Finally, we corrected the Beddington–DeAngelis interference term to reflect that interference cannot occur when only one daphnid is present (i.e. *C* = 0.0033).

We added a third trophic level to Equation 1 to describe the interacting populations of algae, daphnia and hydra (*P*, individuals mL^−1^):

(2A) $$\frac{\mathrm{dR}}{\mathrm{dt}}=\mathrm{rR}\left(1-R*{\mathrm{Qe}}^{\mathrm{Bt}}\right)-\frac{a_{c}\mathrm{RC}}{1+a_{c}h_{c}R+w_{c}\left(C-0.0033\right)}$$

(2B) $$\frac{\mathrm{dC}}{\mathrm{dt}}=\frac{e_{c}a_{c}\mathrm{RC}}{1+a_{c}h_{c}R+w_{c}\left(C-0.0033\right)}-\frac{a_{p}\mathrm{CP}}{1+a_{p}h_{p}C+w_{p}\left(P-0.0033\right)}-d_{c}C$$

(2C) $$\frac{\mathrm{dP}}{\mathrm{dt}}=\frac{e_{p}a_{p}\mathrm{CP}}{1+a_{p}h_{p}C+w_{p}\left(P-0.0033\right)}-d_{p}P$$

Parameters in Equation 2 match those in Equation 1, with the following additions: *a*_*p*_ is the hydra space clearance rate on daphnia, *h*_*p*_ is the handling time, *w*_*p*_ is interference among hydra, *e*_*p*_ is the efficiency of converting daphnia into new hydra, and *d*_*p*_ is the hydra background death rate. As in Equation 1, we corrected the Beddington–DeAngelis interference term to ensure that interference does not occur when only one predator is present.

### 2.3 Model fitting

We used a Bayesian approach to fit Equations 1 and 2 to our two‐ and three‐species time series, respectively. We estimated model parameters using Hamiltonian Monte Carlo sampling in Stan (Version 2.32.2), via the RStan package (Version 2.23.6; Stan Development Team, 2024) in R (Version 4.4.1; R Core Team, 2024). We used a complete pooling approach as described in Rosenbaum et al. (2019). Specifically, we estimated a single set of parameters for each temperature and community combination, and only the initial states varied for each replicate (*m*) time series within a treatment (see Supporting Information S1B). This approach assumes that foraging and demographic rates are shared across replicates within a treatment, while allowing replicate‐specific dynamics to emerge from differences in initial conditions. Complete pooling improves parameter identifiability when individual replicate time series are insufficient to constrain complex ODE models, as was the case here. We fit models separately to each temperature–treatment combination, yielding posterior distributions for each parameter in each temperature–treatment combination (see Supporting Information S1). To constrain estimates to biologically plausible ranges, we set a lower bound of 0 for all estimated parameters and an upper bound for most (see Supporting Information Tables A1 and 2). We chose upper bounds based on conservative biological reasoning, such that parameter values beyond these limits would imply implausible rates or efficiencies given the natural history of the study system.

We first fit Equation 1 to the algae (resource) and daphnia (consumer) time series. For the two‐species treatments, we used weakly informative priors and ran three MCMC chains in parallel with a warmup phase of 3000–5000 iterations followed by 5000 sampling iterations, yielding 15,000 posterior samples across the three chains. For the 17°C treatment, we narrowed priors using the mean and standard deviations from the output of the more dynamic warmer 20°C fits because broad, weakly informed priors produced poor fits to the data at 17°C. We applied the same approach to the 14°C, using the mean and standard deviation from the output of the 17°C to inform priors. A full list of priors for the two‐species fits is provided in Supporting Information Tables B1–B5.

We fit Equation 2 to the three‐species time series, running three MCMC chains in parallel with a warmup phase of 5000 iterations and a sampling phase of 5000 iterations, yielding 15,000 samples approximating the posterior distribution across the three chains. For these fits, we initially fixed parameters shared with Equation 1 (*r, Q, a*_*c*_, *e*_*c*_, *h*_*c*_, *d*_*c*_, *w*_*c*_, *B*) at the median values of the posterior distribution from the corresponding temperature treatment of the two‐species fits and estimated only top predator parameters (*a*_*p*_, *e*_*p*_, *h*_*p*_, *d*_*p*_, *w*_*p*_). However, fixing shared parameters did not generate quality fits of Equation 2 to the data, indicating that daphnia foraging and algae growth parameters needed to be re‐estimated to capture system dynamics in the presence of hydra. We therefore sequentially un‐fixed the Equation 1 parameters (i.e. allowed them to be fit) until we achieved a good fit that displayed convergence between MCMC chains and had high effective sample sizes. We first un‐fixed daphnia foraging parameters (*a*_*c*_, *w*_*c*_, *h*_*c*_), as a priori we would expect changes to the consumer foraging behaviour when foraging under risk (Spitze, 1991; Wojtal‐Frankiewicz, 2012). We then unfixed algae growth parameters (*r, Q*), as we expected their life histories to change as the trophic cascade altered their consumption risk (Chase, 1999; De Roos & Persson, 2002; Fisher et al., 2016; Reznick & Endler, 1982; Rober et al., 2022; Walsh & Reznick, 2008). We also evaluated models that allowed additional shared parameters (*d*_*c*_, *e*_*c*_, *B*) to vary; however, doing so did not improve model fit or convergence and substantially increased model dimensionality, so these parameters were retained as fixed. For these unfixed parameters, we used the estimated means and standard deviations from the corresponding two‐species fits as priors, apart from *a*_*c*_, which required a prior with a higher mean and standard deviation to achieve good fits to the data across all temperature treatments. Using the two‐species fits to inform priors for shared parameters in the three‐species model does not constrain estimates to match predator‐free dynamics. Rather, these priors were used as informative starting points to reflect biologically reasonable magnitudes and to improve numerical stability in the higher‐dimensional model. We used weakly informative priors to fit parameters exclusive to Equation 2 (*a*_*p*_, *e*_*p*_, *h*_*p*_, *d*_*p*_, *w*_*p*_). See Supporting Information Tables B6–B10 for a complete list of priors used to fit three‐species time series.

We obtained good fits to the three‐species time series only when most parameters were not fixed. These included the parameters *a*_*p*_, *e*_*p*_, *h*_*p*_, *d*_*p*_, *w*_*p*_, *r, Q, a*_*c*_, *h*_*c*_ and *w*_*c*_. In contrast, *e*_*c*_, *d*_*c*_, and *B* remained fixed at the median values from the corresponding temperature of the two‐species posterior distributions and did not need to be changed from the two‐species fits to generate a quality match to the data. Visual inspection of trace plots, posterior densities and Gelman–Rubin statistics confirmed that MCMC chains were well mixed and had converged ($\hat{R}<1.01$). We also verified that effective sample sizes were adequate. See Supporting Information Tables C1–C10 for a complete list of summary statistics and Figures A1–A10 for trace plots for each fit.

We assessed differences in parameter estimates between treatments by examining shifts in posterior distributions across temperatures and food chain lengths. The breadth of the posterior distributions reflects not just variation across replicate mesocosms but also the uncertainty in their estimation. Here, we focus on describing how the posterior distributions shift across temperature and between the two‐ and three‐trophic level models.

### 2.4 Calculating trophic cascade strengths

We quantified trophic cascade strength as the ratio of the algae abundance (*R*) in the presence of the predator (*R*_3_, three‐trophic levels) to the algae abundance without the predator (*R*_2_, two trophic levels; Borer et al., 2005; DeLong et al., 2015; Shurin & Seabloom, 2005):

(3) $${\mathrm{TC}}_{\text{IS}}=\frac{R_{3}}{R_{2}}$$

To calculate trophic cascade strength, we solved both the two‐ and three‐species ODEs using parameter sets drawn from posterior distributions for each temperature treatment. For both models, we used all available iterations, with each iteration consisting of a complete set of jointly sampled parameters. We averaged initial values across replicates for each iteration. This yielded 15,000 ODE solutions per temperature–community combination. We then calculated the strength of the trophic cascade at each time step of the ODE (the times at which explicit estimates of *P, C*, and *R* are calculated by the solver) by dividing resource abundances in the predator‐present system (*R*_3_) by the resource abundances in the predator‐absent system (*R*_2_) for each temperature treatment (Equation 3). We then averaged trophic cascade strength across the time series for each of the 15,000 trophic cascades, generating a posterior distribution of 15,000 mean trophic cascade strengths per temperature. Finally, to evaluate how cascade strength responded to warming, we fit linear regressions to sets of single randomly drawn posterior estimates of mean trophic cascade strength from each temperature, producing 15,000 estimates of the slope. This process approximated a posterior distribution of the linear relationship between temperature and mean trophic cascade strength (see Supporting Information S4, Figure B1).

## 3 RESULTS

### 3.1 Population dynamics

**FIGURE 1:**
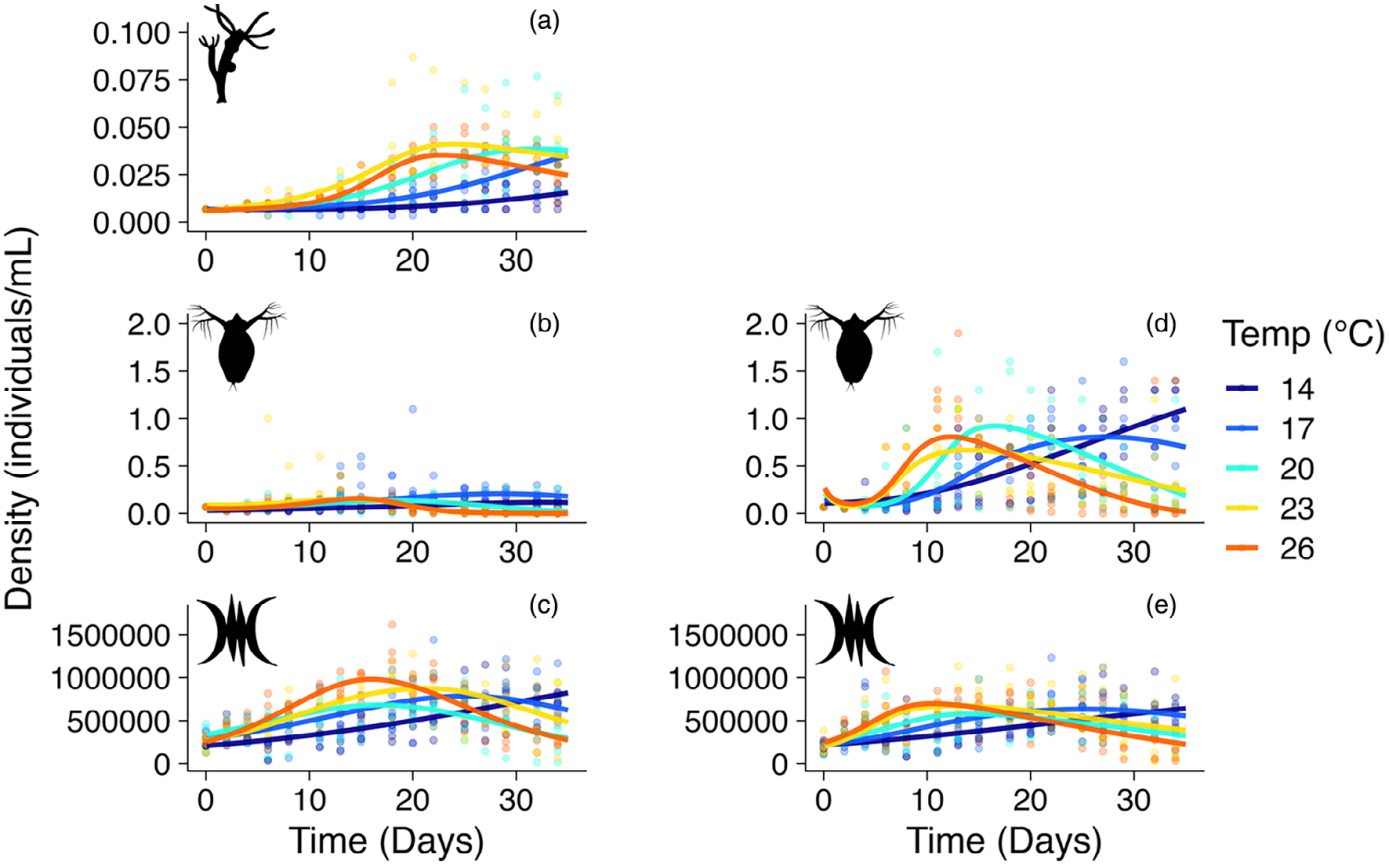
Dynamics of interacting hydra, daphnia and algae populations. Points are replicate‐level population densities on each sampling day. Lines are the posterior predictive mean of ODE solutions calculated from parameter values sampled across posterior distributions (Equations 1 and 2; Figure 3). Temperatures are shown from cool to warm colours. Panels (a–c) show dynamics of the system in the presence of hydra predators. Panels (d and e) show dynamics in the absence of hydra.

The dynamics of interacting daphnia and algae populations differed in the presence and absence of hydra (Figure 1). Hydra reduced daphnia abundances (Figure 1b,d), which in turn increased algae densities by releasing them from herbivory pressure—a trophic cascade (c.f. Figure 1c,e). These dynamics also shifted with temperature. Hydra populations grew faster at warmer temperatures, leading to stronger suppression of daphnia and higher algae densities with warming (Figure 1a–c). Across treatments, all taxa displayed shorter population fluctuation periods and faster growth with warming. At cooler temperatures (14°–17°C), population abundances increased gradually and did not exhibit pronounced peaks over the experimental period, possibly indicating incomplete population cycles. Populations at 20°C and above reached a peak followed by a decline over the course of the timeseries (Figure 1).

### 3.2 Trophic cascade strength

**FIGURE 2:**
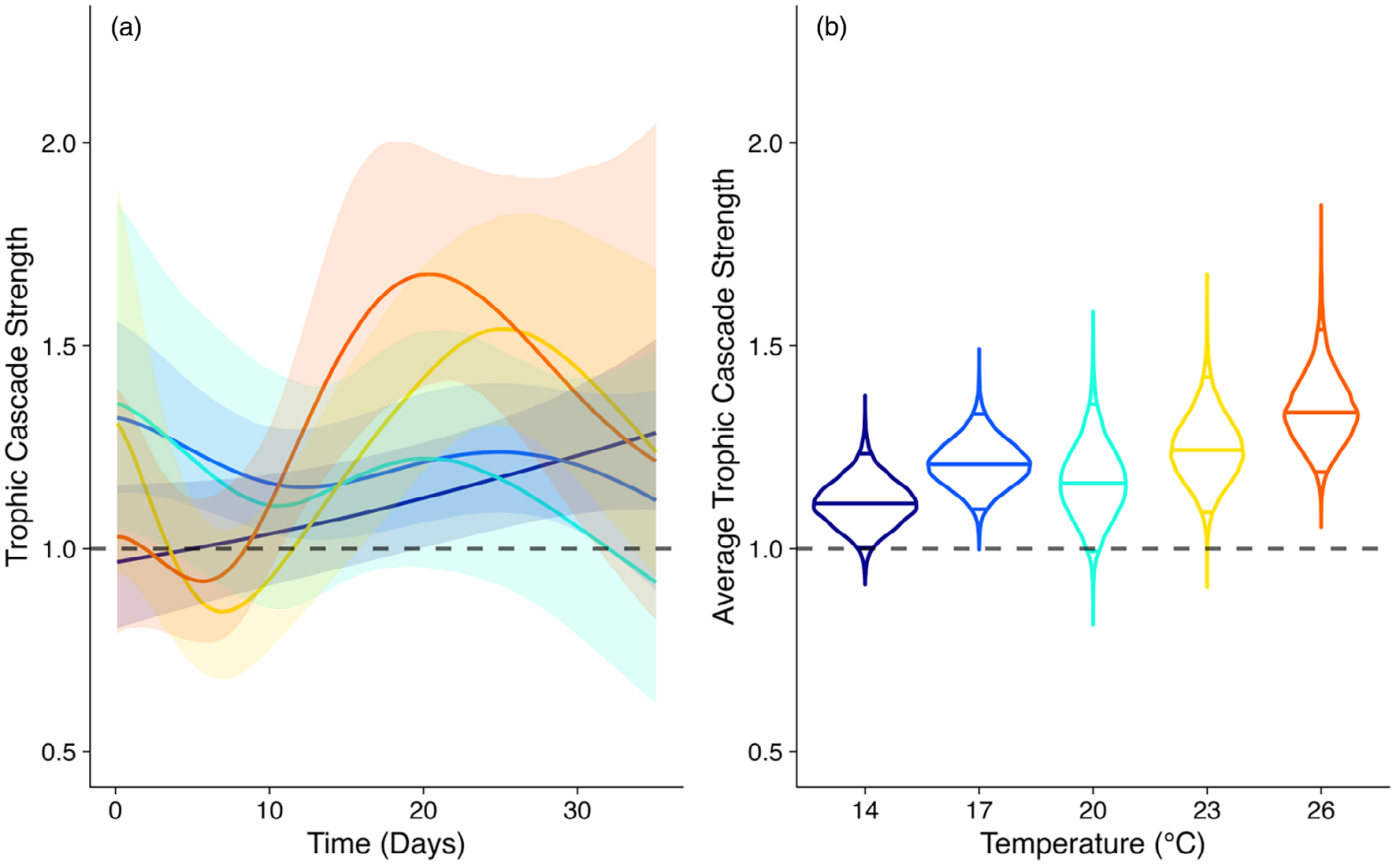
Strength of observed trophic cascades across temperatures. Panel (a) shows the strength of trophic cascades through time. Trophic cascade strength is defined as the ratio of algae abundance (*R*) in the presence of the predator (*R*_3_) to the algae abundance without the predator (*R*_2_) ($\frac{R_{3}}{R_{2}}$). Shaded regions represent 95% credible intervals. Panel (b) shows the distributions of trophic cascade strengths across temperature treatments averaged through for each estimate of the trophic cascade through time. Horizontal lines in violin plots show 2.5%, 50% and 97.5% quantiles. Dashed lines show values where no trophic cascade is observed (i.e. $\frac{R_{3}}{R_{2}}$ = 1).

Trophic cascade strength varied more over time as temperature increased (Figure 2a). Cooler treatments (14–17°C) showed milder variation in trophic cascade strength, while warmer treatments (23–26°C) exhibited stronger and more variable cascades, especially between days 15–25 (Figure 2a). The 20°C treatment was intermediate, overlapping with both cooler and warmer temperatures, and thus appeared to represent a transition point between the two regimes. Overall, trophic cascades were weaker but more consistent at 14–20°C and stronger but more variable at 23–26°C. At warmer temperatures, cascades were initially weak (days 0–10) but intensified later (days 15–30; Figure 2a).

Time‐averaged trophic cascade strength increased with warming, as shown by the distributions of the ratio of the fitted $R_{3}$ to the fitted $R_{2}$ shifting upward with temperatures (Figure 2b). The complementary linear regression analysis estimated a probability of 0.99 that the slope relating mean trophic cascade to temperature was positive, indicating that average trophic cascade strength increased with warming (see Supporting Information S4, Figure B1).

### 3.3 Model parameters

**FIGURE 3:**
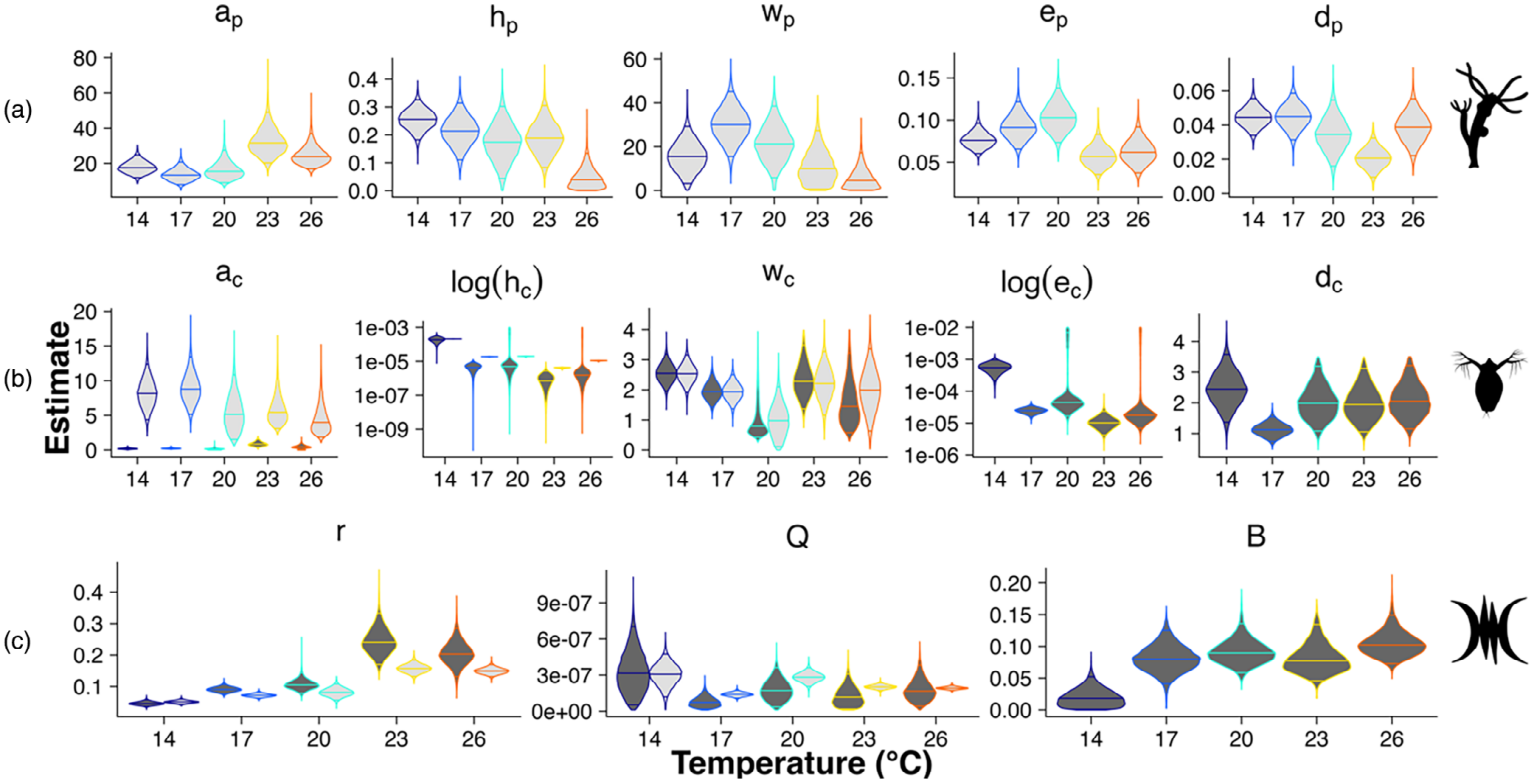
Posterior distributions of estimated parameters from model fits. Row (a) shows parameters associated with hydra feeding, growth and mortality. Row (b) shows parameters associated with daphnia feeding, growth and mortality. Row (c) shows parameters associated with logistic growth in algae. Horizontal lines in violin plots show 2.5%, 50% and 97.5% quantiles. Darker filled violin plots show posterior distributions from treatments without hydra, while lighter filled violin plots show posterior distributions from treatments with hydra. Parameters *h*_*c*_ and e_c_ were plotted on the log_10_ scale.

The posterior distributions of several parameters shared between the two‐ and three‐species models differed across food chain lengths (Figure 3). For example, daphnia space clearance rates (*a*_*c*_) were higher in the presence of hydra, while daphnia handling time (*h*_*c*_) and interference (*w*_*c*_) were unaffected by the presence of hydra (Figure 3b). Algae maximum population growth rates (*r*) were lower when hydra were present, whereas algae intraspecific density dependence (*Q*) did not differ between predator treatments (Figure 3c).

Parameter responses to temperature also varied. Hydra space clearance rates (*a*_*p*_) increased from cooler to warmer temperatures, with a slight decline from 23 to 26°C, while hydra handling time (*h*_*p*_) decreased from 14 to 20°C, remained constant from 20 to 23°C and dropped sharply at 26°C. Hydra interference (*w*_*p*_) and conversion efficiency (*e*_*p*_) exhibited unimodal responses to temperature, with hydra interference peaking at 17° and hydra conversion efficiency peaking at 20°C. Hydra death rates (*d*_*p*_) were stable at 14 to 17°C, decreased until 23°C and then increased again at 26°C (Figure 3a).

Daphnia parameters also shifted with temperature. Daphnia handling time (*h*_*c*_) was highest at 14°C and lower between 17 and 26°C. Daphnia interference (*w*_*c*_) decreased from 14 to 20°C, increased from 20 to 23°C and remained constant through 26°C regardless of hydra presence. Daphnia conversion efficiency (*e*_*c*_) was highest at 14°C and lower at 17 to 26°C. Daphnia maximum death rates (*d*_*c*_) were lowest at 17°C, highest at 14°C, and similar from 20 to 26°C (Figure 3b). Algal parameters showed further responses. Algae growth rate (*r*) increased with warming until 23°C and declined slightly at 26°C. The per capita intraspecific competition (*Q*) was highest at 14°C and consistently lower at 17 to 26°C (Figure 3c).

Finally, in some cases, temperature responses differed between the two‐ and three‐species models. In particular, daphnia space clearance rates (*a*_*c*_) increased with temperature in the absence of hydra but followed a unimodal response in the presence of hydra, peaking at 17°C before declining at higher temperatures (Figure 3b).

## 4 DISCUSSION

Predicting how warming alters trophic cascades requires an understanding of how temperature shapes the links that transmit predator effects to producers through intermediary consumers. While temperature effects on pairwise predator–prey interactions in ectotherms are well established (Bannerman et al., 2011; Burnside et al., 2014; DeLong & Lyon, 2020; Gilbert et al., 2014; Meisner et al., 2014; Rall et al., 2010; Savage et al., 2004; Uiterwaal & DeLong, 2020; Uszko et al., 2017), it remains unclear how temperature might affect the net strength of linked consumer–resource interactions and how these changes, together with temperature‐dependent demographic processes, affect the overall strength of a trophic cascade. Here, we used a combination of theoretical and empirical approaches to characterize the dynamics of a tri‐trophic system and measure the trophic cascade strength across a temperature gradient. We found that warming accelerated system dynamics, producing faster oscillations in abundances (Figure 1) and promoted stronger trophic cascades transiently that ultimately yielded higher average trophic cascade strengths with warming (Figure 2). These temperature‐sensitive trophic cascade strengths arose from a varied suite of temperature sensitivities in the interactions between consumer–resource pairs and their demography (Figure 3). Together, our findings support the hypothesis that warming can increase the strength of trophic cascades, and our results highlight that outcomes depend on multiple interacting mechanisms and may vary substantially through time.

Our results suggest that warming simultaneously facilitates and impedes strong trophic cascades by altering multiple temperature‐dependent biological processes. Increases in hydra space clearance rate (*a*_*p*_) and algae growth rate (*r*) combined with decreases in hydra handling time (*h*_*p*_) and algae intraspecific competition (*Q*) with warming strengthened the trophic cascade. The increase in hydra space clearance rate and decrease in hydra handling time are consistent with the well‐documented pattern that predators tend to be more efficient foragers at higher temperatures, potentially due to increased prey encounter rates and faster prey processing and digestion as environmental temperature rises (DeLong & Lyon, 2020; Englund et al., 2011; Rall et al., 2010). Similarly, increased algal growth rates together with reduced intraspecific competition are consistent with prior findings that primary producers often exhibit faster population growth under warmer conditions (Bieg & Vasseur, 2024; Fan et al., 2014; Lürling et al., 2013). Collectively, these changes strengthened trophic cascades by amplifying the direct negative effects of hydra on daphnia and of daphnia on algae, thereby increasing the strength of trophic linkages through which cascading effects propagate.

In contrast, other temperature‐driven changes acted to weaken trophic cascades, although these effects were outweighed by the strengthening mechanisms described above. Decreases in daphnia space clearance rate (*a*_*c*_) and daphnia conversion efficiency (*e*_*c*_) decreased the direct effect strengths with warming, reducing the ability for the trophic cascade to propagate across trophic levels. The decline in daphnia space clearance rate is consistent with previously documented temperature responses over similar thermal temperatures (West & Post, 2016). Moreover, our mechanistic interpretation is consistent with previous work suggesting that decreases in conversion efficiency can weaken direct effects and dampen trophic cascade strengths (Zhou et al., 2025). While these processes dampen consumer impacts on lower trophic levels, their effects were weaker than the concurrent increases in predator foraging efficiency and algal growth, leading to a net strengthening of the trophic cascade with warming.

Finally, some processes exhibited non‐linear responses to temperature: hydra interference (*w*_*p*_) and hydra conversion efficiency (*e*_*p*_) responded unimodally to temperature, while daphnia interference (*w*_*c*_) and hydra mortality (*d*_*p*_) showed u‐shaped responses, thus having non‐linear effects on trophic cascade strength with warming (Figure 3). Such non‐linearities are consistent with predictions that many physiological and behavioural traits peak at intermediate temperatures before declining as organisms experience thermal or energetic constraints at warmer extremes (Sentis et al., 2012; Uiterwaal & DeLong, 2020; Uszko et al., 2017).

We speculate that these non‐linear responses had more complex and partially opposing effects on trophic cascade strength. For example, hydra interference and conversion efficiency peaked at intermediate temperatures, while hydra mortality and daphnia interference were lowest at similar temperatures but increased at both cooler and warmer extremes (Figure 3). As a result, the effects of these parameters on trophic cascade strength may partially offset one another across the temperature gradient, reducing their net contribution relative to monotonic changes in predator foraging efficiency and algal growth. Together, these results suggest that warming influences trophic cascades through the combined outcome of multiple processes with distinct and sometimes non‐linear temperature dependencies, rather than through any single mechanism.

Our results also indicate that the strength of a trophic cascade might not be predictable from independently estimating pairwise interactions. The ODE fitting analysis revealed that the presence of hydra modified the strength of the daphnia–algae interaction through non‐consumptive effects on daphnia foraging and algae demography as reflected in shifts in the posterior distributions of those parameters. In particular, the presence of hydra greatly increased daphnia space clearance rate, strengthening the direct effect of daphnia on algae (Figure 3b). At first glance, the increase in daphnia space clearance rate in the presence of a predator may appear counterintuitive, as prey are often expected to reduce foraging effort under predation risk (Spitze, 1991; Wojtal‐Frankiewicz, 2012). However, we observed that daphnia exposed to hydra tended to occupy lower regions of the mesocosms, potentially as a predator‐avoidance response, where algal cells were concentrated due to settling. This spatial redistribution likely increased encounter rates between daphnia and algae, leading to higher effective space clearance rates despite increased predation risk. Such spatially mediated responses are consistent with previous work showing that concentrating consumers and resources within part of an arena can enhance clearance rates (Uiterwaal et al., 2019). Furthermore, these outcomes represent a form of interaction modification through non‐consumptive, behaviourally mediated effects, implying that non‐consumptive effects may strongly shape trophic cascades in some systems (Coblentz et al., 2024; Palmer et al., 2022; Ripple & Beschta, 2004; Start & Gilbert, 2017).

More surprisingly, hydra presence altered the temperature dependence of the daphnia–algae interaction (Figure 3b,c). In the absence of hydra, daphnia clearance rate was relatively low but increased with temperature. In contrast, when hydra were present, daphnia space clearance rate was elevated overall but declined with temperature, effectively reversing the temperature dependence of this key consumer–resource interaction (Figure 3; Figure C4). Although the precise mechanisms underlying this shift are difficult to isolate, a plausible explanation emerges from the coupled temperature responses of predator and prey foraging traits. Hydra space clearance rates increased and handling times decreased with warming, indicating that predation pressure on daphnia intensified at higher temperatures. Under these conditions, daphnia may increasingly reduce foraging activity as temperatures rise in the presence of hydra, consistent with behavioural responses to elevated predation risk (Spitze, 1991; Wojtal‐Frankiewicz, 2012). Thus, the initially high daphnia clearance rates observed under predator presence may reflect spatial or behavioural shifts at cooler temperatures, while the subsequent decline with warming likely reflects escalating predator efficiency and risk. These findings demonstrate how predation can restructure food chain interactions through cascading effects (Baker et al., 2025) and how such shifts in species interactions can indirectly modify the effects of warming on food web dynamics (O'Gorman et al., 2023). Together, this suggests that the effects of warming on trophic cascades are highly contingent on community structure.

Our results show that warming increases both the magnitude and temporal variability of trophic cascade strength, with transient peaks in cascade strength emerging at warmer temperatures that elevate the time‐averaged cascade effect. Although trophic cascades are often thought of as a phenomenon with a fixed magnitude (Shanafelt & Loreau, 2018), trophic cascades can vary substantially though time rather than occurring only at equilibrium (Langendorf et al., 2025). Such temporal variation arises from ecological, environmental and phenological factors that alter the strength of direct effects and population abundances through time (Bridgeland et al., 2010; Piovia‐Scott et al., 2017; Post et al., 1999; Preisser & Strong, 2004). Warming, in particular, can amplify this variation by making systems more dynamic (Barton et al., 2009; DeLong & Lyon, 2020; Meisner et al., 2014; Nelson et al., 2013; Yan et al., 2013). Our results provide clear evidence that higher temperatures intensified the transient strength of trophic cascades. Our system showed strong dynamic responses to shifts in environmental temperature (Figure 2b) driven by a net increase in the strength of direct effects with warming, resulting in faster and more oscillatory dynamics at warmer temperatures (Figures 1 and 3). These oscillations, particularly in resource dynamics, generated episodic spikes in trophic cascade strength at warmer temperatures. Although cascade strength was more variable, these transient peaks resulted in greater average cascade strength in warmer systems than in cooler, more weakly dynamic systems. (Figures 1c,e and 2). This suggests that rising temperatures can enhance the transient effects of predators on resources beyond what would be predicted by equilibrium or average effects. Such dynamics may be especially important in systems with low resilience (i.e. systems vulnerable to changes in resource abundances) or systems with high variability in species abundances where transient spikes in indirect effect strengths could have disproportionate consequences for community stability (Hastings et al., 2018).

Although our findings come from a laboratory model system, they highlight general mechanisms by which warming can alter interaction strengths in ectotherm food webs, even if the magnitude and direction of these effects may vary across systems. The observed temperature dependencies of hydra space clearance rate and handling time in the three‐species treatment and daphnia space clearance rates in the two‐species treatment align with previous work demonstrating that warming generally increases space clearance rates and decreases handling times (Burnside et al., 2014; Englund et al., 2011; Rall et al., 2012; Uiterwaal & DeLong, 2020). The warming‐driven increase in daphnia space clearance rates also mirrors previous work demonstrating that elevated temperatures strengthen the direct effects of herbivores on algae (O'Connor, 2009). Likewise, previous studies have also demonstrated that per capita growth rates are temperature‐dependent in *Ankistrodesmus* (Safarov et al., 2015) and other single‐celled green algae species (Singh & Singh, 2015). While some features of our system, such as the hydra‐mediated increase in daphnia clearance rates, may reflect the idiosyncrasies of our system and laboratory environment, it is reasonable to expect that other systems containing ectotherms may experience similar changes in direct effect strengths through warming‐induced shifts in consumer foraging and resource demographics, with comparable consequences for trophic interactions.

Furthermore, temporal changes in consumer population structure may also contribute to variation in trophic cascade strength through time. Our analysis treated daphnia as an unstructured population, but shifts in size or age distributions can strongly influence both grazing rates on algae and vulnerability to hydra predation (Davidson et al., 2024; Riessen, 1999; Spitze, 1991). Because our experiment and modelling framework did not explicitly track or parameterize size‐ or stage‐structured dynamics, we cannot disentangle these demographic effects from the behavioural and physiological mechanisms inferred here. Incorporating population structure into future experiments and models will be important for resolving how warming, predation and consumer demography jointly shape the transient and long‐term dynamics of trophic cascades.

Overall, our study provides a mechanistic explanation for how warming can influence trophic cascade strength through temperature‐dependent shifts in interaction traits, a perspective that has been largely missing from the existing literature on the temperature dependence of trophic cascades. By linking temperature‐dependent shifts in predator, prey and resource traits to changes in population dynamics, we demonstrate how these processes ultimately impact the trophic cascade strength experienced across a thermal gradient. Furthermore, our findings highlight the importance of accounting for warming‐driven increases in the temporal variation of trophic cascades, particularly in transient systems where population dynamics can strongly shape community trajectories. Although further work is needed to assess the generality of these patterns, especially in terrestrial and endotherm‐dominated systems and in more complex food webs, our results illustrate how warming can strengthen trophic cascades by modifying the direct effects that link species within an interaction chain.

## AUTHOR CONTRIBUTIONS

Francis P. Biagioli and John P. DeLong designed the study with input from the other authors. Francis P. Biagioli, Liuqingqing Yang, and Dinelka Thilakarathne performed the study. Francis P. Biagioli performed the statistical analyses with input from Kyle E. Coblentz and John P. DeLong. Francis P. Biagioli led the writing of the manuscript; all authors helped to write the manuscript or contributed to revisions.

## CONFLICT OF INTEREST STATEMENT

The authors declare no conflicts of interest.

## STATEMENT OF INCLUSION

Our study brings together authors and collaborators from several countries, including individuals from the United States where the study was carried out. All authors were engaged early on in the study, and their perspectives were considered in research, study design and writing of the manuscript.

## Supporting information


**Table A1.** Upper and lower bounds for estimated parameters used in the ODE model fitted to time series data in the absence of *Hydra*.
**Table A2.** Upper and lower bounds for estimated parameters used in the ODE model fitted to time series data in the presence of *Hydra*.
**Table B1.** Parameter values $\left(\mu \;\text{and}\;\sigma \right)$ for prior distributions used in the ODE model fitted to time series data at 14°C in the absence of *Hydra*.
**Table B2.** Parameter values $\left(\mu \;\text{and}\;\sigma \right)$ for prior distributions used in the ODE model fitted to time series data at 17°C in the absence of *Hydra*.
**Table B3.** Parameter values $\left(\mu \;\text{and}\;\sigma \right)$ for prior distributions used in the ODE model fitted to time series data at 20°C in the absence of *Hydra*.
**Table B4.** Parameter values $\left(\mu \;\text{and}\;\sigma \right)$ for prior distributions used in the ODE model fitted to time series data at 23°C in the absence of *Hydra*.
**Table B5.** Parameter values $\left(\mu \;\text{and}\;\sigma \right)$ for prior distributions used in the ODE model fitted to time series data at 26°C in the absence of *Hydra*.
**Table B6.** Parameter values $\left(\mu \;\text{and}\;\sigma \right)$ for prior distributions used in the ODE model fitted to time series data at 14°C in the presence of *Hydra*.
**Table B7.** Parameter values $\left(\mu \;\text{and}\;\sigma \right)$ for prior distributions used in the ODE model fitted to time series data at 14°C in the presence of *Hydra*.
**Table B8.** Parameter values $\left(\mu \;\text{and}\;\sigma \right)$ for prior distributions used in the ODE model fitted to time series data at 20°C in the presence of *Hydra*.
**Table B9.** Parameter values $\left(\mu \;\text{and}\;\sigma \right)$ for prior distributions used in the ODE model fitted to time series data at 23°C in the presence of *Hydra*.
**Table B10.** Parameter values $\left(\mu \;\text{and}\;\sigma \right)$ for prior distributions used in the ODE model fitted to time series data at 26°C in the presence of *Hydra*.
**Table C1.** Summary statistics for 14°C, two species time series data. Gelman‐Rubin statistics $\hat{R}<1.01$ for all parameters verify convergence, $n_{\mathrm{eff}}$ is the number independent samples.
**Table C2.** Summary statistics for 17°C, two species time series data. Gelman‐Rubin statistics $\hat{R}<1.01$ for all parameters verify convergence, $n_{\mathrm{eff}}$ is the number independent samples.
**Table C3.** Summary statistics for 20°C, two species time series data. Gelman‐Rubin statistics $\hat{R}<1.01$ for all parameters verify convergence, $n_{\mathrm{eff}}$ is the number independent samples.
**Table C4.** Summary statistics for 23°C, two species time series data. Gelman‐Rubin statistics $\hat{R}<1.01$ for all parameters verify convergence, $n_{\mathrm{eff}}$ is the number independent samples.
**Table C5.** Summary statistics for 26°C, two species time series data. Gelman‐Rubin statistics $\hat{R}<1.01$ for all parameters verify convergence, $n_{\mathrm{eff}}$ is the number independent samples.
**Table C6.** Summary statistics for 14°C, three species time series data. Gelman‐Rubin statistics $\hat{R}<1.01$ for all parameters verify convergence, $n_{\mathrm{eff}}$ is the number independent samples.
**Table C7.** Summary statistics for 17°C, three species time series data. Gelman‐Rubin statistics $\hat{R}<1.01$ for all parameters verify convergence, $n_{\mathrm{eff}}$ is the number independent samples.
**Table C8.** Summary statistics for 20°C, three species time series data. Gelman‐Rubin statistics $\hat{R}<1.01$ for all parameters verify convergence, $n_{\mathrm{eff}}$ is the number independent samples.
**Table C9.** Summary statistics for 23°C, three species time series data. Gelman‐Rubin statistics $\hat{R}<1.01$ for all parameters verify convergence, $n_{\mathrm{eff}}$ is the number independent samples.
**Table C10.** Summary statistics for 26°C, three species time series data. Gelman‐Rubin statistics $\hat{R}<1.01$ for all parameters verify convergence, $n_{\mathrm{eff}}$ is the number independent samples.
**Figure A1.** Trace plots of MCMC chains for the two‐species time series data at 14°C. Grey shaded areas indicate warm‐up iterations.
**Figure A2.** Trace plots of MCMC chains for the two‐species time series data at 17°C. Grey shaded areas indicate warm‐up iterations.
**Figure A3.** Trace plots of MCMC chains for the two‐species time series data at 20°C. Grey shaded areas indicate warm‐up iterations.
**Figure A4.** Trace plots of MCMC chains for the two‐species time series data at 23°C. Grey shaded areas indicate warm‐up iterations.
**Figure A5.** Trace plots of MCMC chains for the two‐species time series data at 26°C. Grey shaded areas indicate warm‐up iterations.
**Figure A6.** Trace plots of MCMC chains for the three‐species time series data at 14°C. Grey shaded areas indicate warm‐up iterations.
**Figure A7.** Trace plots of MCMC chains for the three‐species time series data at 17°C. Grey shaded areas indicate warm‐up iterations.
**Figure A8.** Trace plots of MCMC chains for the three‐species time series data at 20°C. Grey shaded areas indicate warm‐up iterations.
**Figure A9.** Trace plots of MCMC chains for the three‐species time series data at 23°C. Grey shaded areas indicate warm‐up iterations.
**Figure A10.** Trace plots of MCMC chains for the three‐species time series data at 26°C. Grey shaded areas indicate warm‐up iterations.
**Figure B1.** Posterior distribution of slope estimates from linear regressions fit to posterior samples of average trophic cascade strength across temperature treatments.
**Figure C1.** Supplements Dynamics of interacting daphnia, and algae populations from two‐species fits.
**Figure C2.** Dynamics of interacting daphnia, and algae populations from three‐species fits.
**Figure C3.** Posterior distribution of parameter estimates related to hydra dynamics from three‐species fits.
**Figure C4.** Posterior distribution of parameter estimates related to daphnia dynamics.
**Figure C5.** Posterior distribution of parameter estimates related to algae dynamics.

## Data Availability

Data available from the Zenodo Digital Repository https://doi.org/10.5281/zenodo.17117255 (Biagioli et al., 2025).

## References

[jane70290-bib-0001] Antiqueira, P. A. P. , Petchey, O. L. , Rezende, F. , Machado Velho, L. F. , Rodrigues, L. C. , & Romero, G. Q. (2022). Warming and top predator loss drive direct and indirect effects on multiple trophic groups within and across ecosystems. Journal of Animal Ecology, 91(2), 428–442. 10.1111/1365-2656.13640 34808001

[jane70290-bib-0002] Atwood, T. B. , Hammill, E. , Greig, H. S. , Kratina, P. , Shurin, J. B. , Srivastava, D. S. , & Richardson, J. S. (2013). Predator‐induced reduction of freshwater carbon dioxide emissions. Nature Geoscience, 6(3), 191–194. 10.1038/ngeo1734

[jane70290-bib-0003] Baker, R. S. , Mott, C. L. , & Whiteman, H. H. (2025). Predation alters community structure through multiple trophic cascades. Journal of Animal Ecology, 94, 1365‐2656.70083. 10.1111/1365-2656.70083 40548831

[jane70290-bib-0004] Bannerman, J. A. , Gillespie, D. R. , & Roitberg, B. D. (2011). The impacts of extreme and fluctuating temperatures on trait‐mediated indirect aphid‐parasitoid interactions. Ecological Entomology, 36(4), 490–498. 10.1111/j.1365-2311.2011.01292.x

[jane70290-bib-0005] Barton, B. T. , Beckerman, A. P. , & Schmitz, O. J. (2009). Climate warming strengthens indirect interactions in an old‐field food web. Ecology, 90(9), 2346–2351. 10.1890/08-2254.1 19769112

[jane70290-bib-0006] Beddington, J. R. (1975). Mutual interference between parasites or predators and its effect on searching efficiency. The Journal of Animal Ecology, 44(1), 331. 10.2307/3866

[jane70290-bib-0007] Berlow, E. L. , Dunne, J. A. , Martinez, N. D. , Stark, P. B. , Williams, R. J. , & Brose, U. (2009). Simple prediction of interaction strengths in complex food webs. Proceedings of the National Academy of Sciences, 106(1), 187–191. 10.1073/pnas.0806823106 PMC2629248 19114659

[jane70290-bib-0008] Biagioli, F. P. , Coblentz, K. E. , & DeLong, J. P. (2025). Warming increases trophic cascade strength code release V4 (version v2.0) [Computer software]. *Zenodo*. 10.5281/ZENODO.17117255

[jane70290-bib-0009] Bieg, C. , & Vasseur, D. (2024). Interactions between temperature and nutrients determine the population dynamics of primary producers. Ecology Letters, 27(1), e14363. 10.1111/ele.14363 38235912

[jane70290-bib-0010] Borer, E. T. , Seabloom, E. W. , Shurin, J. B. , Anderson, K. E. , Blanchette, C. A. , Broitman, B. , Cooper, S. D. , & Halpern, B. S. (2005). What determines the strength of a trophic cascade? Ecology, 86(2), 528–537. 10.1890/03-0816

[jane70290-bib-0011] Bosch, T. C. , Krylow, S. M. , Bode, H. R. , & Steele, R. E. (1988). Thermotolerance and synthesis of heat shock proteins: These responses are present in *Hydra attenuata* but absent in *Hydra oligactis* . Proceedings of the National Academy of Sciences, 85(21), 7927–7931. 10.1073/pnas.85.21.7927 PMC282326 3186697

[jane70290-bib-0012] Brett, M. T. , & Goldman, C. R. (1996). A meta‐analysis of the freshwater trophic cascade. Proceedings of the National Academy of Sciences, 93(15), 7723–7726. 10.1073/pnas.93.15.7723 PMC38814 11607694

[jane70290-bib-0013] Bridgeland, W. T. , Beier, P. , Kolb, T. , & Whitham, T. G. (2010). A conditional trophic cascade: Birds benefit faster growing trees with strong links between predators and plants. Ecology, 91(1), 73–84. 10.1890/08-1821.1 20380198

[jane70290-bib-0014] Burnside, W. R. , Erhardt, E. B. , Hammond, S. T. , & Brown, J. H. (2014). Rates of biotic interactions scale predictably with temperature despite variation. Oikos, 123(12), 1449–1456. 10.1111/oik.01199

[jane70290-bib-0015] Carpenter, S. R. , Brock, W. A. , Cole, J. J. , Kitchell, J. F. , & Pace, M. L. (2008). Leading indicators of trophic cascades. Ecology Letters, 11(2), 128–138. 10.1111/j.1461-0248.2007.01131.x 18021242

[jane70290-bib-0016] Carpenter, S. R. , & Kitchell, J. F. (1988). Consumer control of lake productivity. Bioscience, 38(11), 764–769. 10.2307/1310785

[jane70290-bib-0017] Chase, J. M. (1999). To grow or to reproduce? The role of life‐history plasticity in food web dynamics. The American Naturalist, 154(5), 571–586. 10.1086/303261 10561129

[jane70290-bib-0018] Coblentz, K. E. , Squires, A. , Uiterwaal, S. , & Delong, J. P. (2022). Quantifying predator functional responses under field conditions reveals interactive effects of temperature and interference with sex and stage. Journal of Animal Ecology, 91(7), 1431–1443. 10.1111/1365-2656.13703 35426950 PMC9540483

[jane70290-bib-0019] Coblentz, K. E. , Treidel, L. A. , Biagioli, F. P. , Fragel, C. G. , Johnson, A. E. , Thilakarathne, D. D. , Yang, L. , & DeLong, J. P. (2024). A framework for understanding climate change impacts through non‐compensatory intra‐ and interspecific climate change responses. Global Change Biology, 30(6), e17378. 10.1111/gcb.17378 38923246

[jane70290-bib-0020] Costamagna, A. C. , Landis, D. A. , & Difonzo, C. D. (2007). Suppression of soybean aphid by generalist predators results in a trophic cascade in soybeans. Ecological Applications, 17(2), 441–451. 10.1890/06-0284 17489251

[jane70290-bib-0021] Davidson, A. T. , Stunkle, C. R. , Armstrong, J. T. , Hamman, E. A. , McCoy, M. W. , & Vonesh, J. R. (2024). Warming and top‐down control of stage‐structured prey: Linking theory to patterns in natural systems. Ecology, 105(1), e4213. 10.1002/ecy.4213 38029361

[jane70290-bib-0022] De Roos, A. M. , & Persson, L. (2002). Size‐dependent life‐history traits promote catastrophic collapses of top predators. Proceedings of the National Academy of Sciences, 99(20), 12907–12912. 10.1073/pnas.192174199 PMC130558 12237404

[jane70290-bib-0023] DeAngelis, D. L. , Goldstein, R. A. , & O'Neill, R. V. (1975). A model for tropic interaction. Ecology, 56(4), 881–892. 10.2307/1936298

[jane70290-bib-0024] DeLong, J. P. , Gilbert, B. , Shurin, J. B. , Savage, V. M. , Barton, B. T. , Clements, C. F. , Dell, A. I. , Greig, H. S. , Harley, C. D. G. , Kratina, P. , McCann, K. S. , Tunney, T. D. , Vasseur, D. A. , & O'Connor, M. I. (2015). The body size dependence of trophic cascades. The American Naturalist, 185(3), 354–366. 10.1086/679735 25674690

[jane70290-bib-0025] DeLong, J. P. , & Lyon, S. (2020). Temperature alters the shape of predator–prey cycles through effects on underlying mechanisms. PeerJ, 8, e9377. 10.7717/peerj.9377 32596054 PMC7307560

[jane70290-bib-0026] DeLong, J. P. , & Vasseur, D. A. (2011). Mutual interference is common and mostly intermediate in magnitude. BMC Ecology, 11(1), 1. 10.1186/1472-6785-11-1 21211032 PMC3024213

[jane70290-bib-0027] Englund, G. , Öhlund, G. , Hein, C. L. , & Diehl, S. (2011). Temperature dependence of the functional response. Ecology Letters, 14(9), 914–921. 10.1111/j.1461-0248.2011.01661.x 21752171

[jane70290-bib-0028] Estes, J. A. , Terborgh, J. , Brashares, J. S. , Power, M. E. , Berger, J. , Bond, W. J. , Carpenter, S. R. , Essington, T. E. , Holt, R. D. , Jackson, J. B. C. , Marquis, R. J. , Oksanen, L. , Oksanen, T. , Paine, R. T. , Pikitch, E. K. , Ripple, W. J. , Sandin, S. A. , Scheffer, M. , Schoener, T. W. , … Wardle, D. A. (2011). Trophic downgrading of planet earth. Science, 333(6040), 301–306. 10.1126/science.1205106 21764740

[jane70290-bib-0029] Fan, X. , Xu, D. , Wang, Y. , Zhang, X. , Cao, S. , Mou, S. , & Ye, N. (2014). The effect of nutrient concentrations, nutrient ratios and temperature on photosynthesis and nutrient uptake by Ulva prolifera: Implications for the explosion in green tides. Journal of Applied Phycology, 26(1), 537–544. 10.1007/s10811-013-0054-z

[jane70290-bib-0030] Fisher, R. M. , Bell, T. , & West, S. A. (2016). Multicellular group formation in response to predators in the alga *Chlorella vulgaris* . Journal of Evolutionary Biology, 29(3), 551–559. 10.1111/jeb.12804 26663204

[jane70290-bib-0031] Gilbert, B. , Tunney, T. D. , McCann, K. S. , DeLong, J. P. , Vasseur, D. A. , Savage, V. , Shurin, J. B. , Dell, A. I. , Barton, B. T. , Harley, C. D. G. , Kharouba, H. M. , Kratina, P. , Blanchard, J. L. , Clements, C. , Winder, M. , Greig, H. S. , & O'Connor, M. I. (2014). A bioenergetic framework for the temperature dependence of trophic interactions. Ecology Letters, 17(8), 902–914. 10.1111/ele.12307 24894409

[jane70290-bib-0032] Hastings, A. , Abbott, K. C. , Cuddington, K. , Francis, T. , Gellner, G. , Lai, Y.‐C. , Morozov, A. , Petrovskii, S. , Scranton, K. , & Zeeman, M. L. (2018). Transient phenomena in ecology. Science, 361(6406), eaat6412. 10.1126/science.aat6412 30190378

[jane70290-bib-0033] Holling, C. S. (1959). Some characteristics of simple types of predation and parasitism. The Canadian Entomologist, 91(7), 385–398. 10.4039/Ent91385-7

[jane70290-bib-0034] Huey, R. B. , & Berrigan, D. (2001). Temperature, demography, and ectotherm fitness. The American Naturalist, 158(2), 204–210. 10.1086/321314 18707349

[jane70290-bib-0035] Jarośík, V. , Kratochvíl, L. , Honék, A. , & Dixon, A. F. G. (2004). A general rule for the dependence of developmental rate on temperature in ectothermic animals. Proceedings of the Royal Society of London. Series B: Biological Sciences, 271(suppl_4), S219–S222. 10.1098/rsbl.2003.0145 PMC1810016 15252989

[jane70290-bib-0036] Knight, T. M. , McCoy, M. W. , Chase, J. M. , McCoy, K. A. , & Holt, R. D. (2005). Trophic cascades across ecosystems. Nature, 437(7060), 880–883. 10.1038/nature03962 16208370

[jane70290-bib-0037] Kratina, P. , Greig, H. S. , Thompson, P. L. , Carvalho‐Pereira, T. S. A. , & Shurin, J. B. (2012). Warming modifies trophic cascades and eutrophication in experimental freshwater communities. Ecology, 93(6), 1421–1430. 10.1890/11-1595.1 22834382

[jane70290-bib-0038] Langendorf, R. E. , Estes, J. A. , Watson, J. C. , Kenner, M. C. , Hatfield, B. B. , Tinker, M. T. , Waddle, E. , DeMarche, M. L. , & Doak, D. F. (2025). Dynamic and context‐dependent keystone species effects in kelp forests. Proceedings of the National Academy of Sciences, 122(10), e2413360122. 10.1073/pnas.2413360122 PMC11912371 40030028

[jane70290-bib-0039] Li, C. , Chen, J. , Liao, X. , Ramus, A. P. , Angelini, C. , Liu, L. , Silliman, B. R. , Bertness, M. D. , & He, Q. (2023). Shorebirds‐driven trophic cascade helps restore coastal wetland multifunctionality. Nature Communications, 14(1), 8076. 10.1038/s41467-023-43951-3 PMC10700615 38057308

[jane70290-bib-0040] Lundgren, E. J. , Ramp, D. , Middleton, O. S. , Wooster, E. I. F. , Kusch, E. , Balisi, M. , Ripple, W. J. , Hasselerharm, C. D. , Sanchez, J. N. , Mills, M. , & Wallach, A. D. (2022). A novel trophic cascade between cougars and feral donkeys shapes desert wetlands. Journal of Animal Ecology, 91(12), 2348–2357. 10.1111/1365-2656.13766 35871769 PMC10087508

[jane70290-bib-0041] Lürling, M. , Eshetu, F. , Faassen, E. J. , Kosten, S. , & Huszar, V. L. M. (2013). Comparison of cyanobacterial and green algal growth rates at different temperatures. Freshwater Biology, 58(3), 552–559. 10.1111/j.1365-2427.2012.02866.x

[jane70290-bib-0042] Mallard, F. , Le Bourlot, V. , Le Coeur, C. , Avnaim, M. , Péronnet, R. , Claessen, D. , & Tully, T. (2020). From individuals to populations: How intraspecific competition shapes thermal reaction norms. Functional Ecology, 34(3), 669–683. 10.1111/1365-2435.13516

[jane70290-bib-0043] McCoy, M. W. , & Gillooly, J. F. (2008). Predicting natural mortality rates of plants and animals. Ecology Letters, 11(7), 710–716. 10.1111/j.1461-0248.2008.01190.x 18422635

[jane70290-bib-0044] Meisner, M. H. , Harmon, J. P. , & Ives, A. R. (2014). Temperature effects on long‐term population dynamics in a parasitoid–host system. Ecological Monographs, 84(3), 457–476. 10.1890/13-1933.1

[jane70290-bib-0045] Menge, B. A. (1995). Indirect effects in marine rocky intertidal interaction webs: Patterns and importance. Ecological Monographs, 65(1), 21–74. 10.2307/2937158

[jane70290-bib-0046] Mooney, K. A. , Halitschke, R. , Kessler, A. , & Agrawal, A. A. (2010). Evolutionary trade‐offs in plants mediate the strength of trophic cascades. Science, 327(5973), 1642–1644. 10.1126/science.1184814 20339073

[jane70290-bib-0047] Nelson, W. A. , Bjørnstad, O. N. , & Yamanaka, T. (2013). Recurrent insect outbreaks caused by temperature‐driven changes in system stability. Science, 341(6147), 796–799. 10.1126/science.1238477 23907532

[jane70290-bib-0048] Novak, M. , Yeakel, J. D. , Noble, A. E. , Doak, D. F. , Emmerson, M. , Estes, J. A. , Jacob, U. , Tinker, M. T. , & Wootton, J. T. (2016). Characterizing species interactions to understand press perturbations: What is the community matrix? Annual Review of Ecology, Evolution, and Systematics, 47(1), 409–432. 10.1146/annurev-ecolsys-032416-010215

[jane70290-bib-0049] O'Connor, M. I. (2009). Warming strengthens an herbivore–plant interaction. Ecology, 90(2), 388–398. 10.1890/08-0034.1 19323223

[jane70290-bib-0050] O'Gorman, E. J. , Zhao, L. , Kordas, R. L. , Dudgeon, S. , & Woodward, G. (2023). Warming indirectly simplifies food webs through effects on apex predators. Nature Ecology & Evolution, 7(12), 1983–1992. 10.1038/s41559-023-02216-4 37798434 PMC10697836

[jane70290-bib-0051] Otto, S. B. , Berlow, E. L. , Rank, N. E. , Smiley, J. , & Brose, U. (2008). Predator diversity and identity drive interaction strength and trophic cascades in a food web. Ecology, 89(1), 134–144. 10.1890/07-0066.1 18376555

[jane70290-bib-0052] Paine, R. T. (1980). Food webs: Linkage, interaction strength and community infrastructure. The Journal of Animal Ecology, 49(3), 666. 10.2307/4220

[jane70290-bib-0053] Palmer, M. S. , Gaynor, K. M. , Becker, J. A. , Abraham, J. O. , Mumma, M. A. , & Pringle, R. M. (2022). Dynamic landscapes of fear: Understanding spatiotemporal risk. Trends in Ecology & Evolution, 37(10), 911–925. 10.1016/j.tree.2022.06.007 35817684

[jane70290-bib-0054] Piovia‐Scott, J. , Yang, L. H. , & Wright, A. N. (2017). Temporal variation in trophic cascades. Annual Review of Ecology, Evolution, and Systematics, 48(1), 281–300. 10.1146/annurev-ecolsys-121415-032246

[jane70290-bib-0055] Post, E. , Peterson, R. O. , Stenseth, N. C. , & McLaren, B. E. (1999). Ecosystem consequences of wolf behavioural response to climate. Nature, 401(6756), 905–907. 10.1038/44814

[jane70290-bib-0056] Preisser, E. L. , & Strong, D. R. (2004). Climate affects predator control of an herbivore outbreak. The American Naturalist, 163(5), 754–762. 10.1086/383620 15122492

[jane70290-bib-0057] R Core Team . (2024). R: A language and environment for statistical computing (Version 4.4.1) [Computer software]. R Foundation for Statistical Computing. https://www.R‐project.org/

[jane70290-bib-0058] Rall, B. C. , Brose, U. , Hartvig, M. , Kalinkat, G. , Schwarzmüller, F. , Vucic‐Pestic, O. , & Petchey, O. L. (2012). Universal temperature and body‐mass scaling of feeding rates. Philosophical Transactions of the Royal Society, B: Biological Sciences, 367(1605), 2923–2934. 10.1098/rstb.2012.0242 PMC3479751 23007080

[jane70290-bib-0059] Rall, B. C. , Vucic‐Pestic, O. , Ehnes, R. B. , Emmerson, M. , & Brose, U. (2010). Temperature, predator–prey interaction strength and population stability. Global Change Biology, 16(8), 2145–2157. 10.1111/j.1365-2486.2009.02124.x

[jane70290-bib-0060] Rebolledo, A. P. , Sgrò, C. M. , & Monro, K. (2020). Thermal performance curves reveal shifts in optima, limits, and breadth in early life. Journal of Experimental Biology, 223, jeb.233254. 10.1242/jeb.233254 33071221

[jane70290-bib-0061] Reznick, D. , & Endler, J. A. (1982). The impact of predation on life history evolution in Trinidadian guppies (*Poecilia reticulata*). Evolution, 36(1), 160–177. 10.2307/2407978 28581096

[jane70290-bib-0062] Riessen, H. P. (1999). Predator‐induced life history shifts in *Daphnia*: A synthesis of studies using meta‐analysis. Canadian Journal of Fisheries and Aquatic Sciences, 56(12), 2487–2494. 10.1139/f99-155

[jane70290-bib-0063] Ripple, W. J. , & Beschta, R. L. (2004). Wolves and the ecology of fear: Can predation risk structure ecosystems? Bioscience, 54(8), 755. 10.1641/0006-3568(2004)054[0755:WATEOF]2.0.CO;2

[jane70290-bib-0064] Ripple, W. J. , Beschta, R. L. , Wolf, C. , Painter, L. E. , & Wirsing, A. J. (2025). The strength of the Yellowstone trophic cascade after wolf reintroduction. Global Ecology and Conservation, 58, e03428. 10.1016/j.gecco.2025.e03428

[jane70290-bib-0065] Ripple, W. J. , Estes, J. A. , Schmitz, O. J. , Constant, V. , Kaylor, M. J. , Lenz, A. , Motley, J. L. , Self, K. E. , Taylor, D. S. , & Wolf, C. (2016). What is a trophic cascade? Trends in Ecology & Evolution, 31(11), 842–849. 10.1016/j.tree.2016.08.010 27663836

[jane70290-bib-0066] Ripple, W. J. , Whalen, D. N. , Wolf, C. , Cao, Y. , Schulte, J. , Swann, S. , Woodrich, S. T. , Newsome, T. , Cairncross, R. , & Wirsing, A. J. (2024). Trophic cascades and climate change. Food Webs, 41, e00362. 10.1016/j.fooweb.2024.e00362

[jane70290-bib-0067] Rober, A. R. , McCann, K. S. , Turetsky, M. R. , & Wyatt, K. H. (2022). Cascading effects of predators on algal size structure. Journal of Phycology, 58(2), 308–317. 10.1111/jpy.13235 35032342

[jane70290-bib-0068] Rodríguez‐Castañeda, G. (2013). The world and its shades of green: A meta‐analysis on trophic cascades across temperature and precipitation gradients. Global Ecology and Biogeography, 22(1), 118–130. 10.1111/j.1466-8238.2012.00795.x

[jane70290-bib-0069] Rosenbaum, B. , Raatz, M. , Weithoff, G. , Fussmann, G. F. , & Gaedke, U. (2019). Estimating parameters from multiple time series of population dynamics using Bayesian inference. Frontiers in Ecology and Evolution, 6, 234. 10.3389/fevo.2018.00234

[jane70290-bib-0070] Safarov, I. , Abdullaev, A. , Khujamshukurov, N. , & Shakirov, Z. (2015). Influence of temperature and CO_2_ on the growth and accumulation oil of microalgae. British Journal of Applied Science & Technology, 10(3), 1–9. 10.9734/BJAST/2015/18018

[jane70290-bib-0071] Savage, V. M. , Gillooly, J. F. , Brown, J. H. , West, G. B. , & Charnov, E. L. (2004). Effects of body size and temperature on population growth. The American Naturalist, 163(3), 429–441. 10.1086/381872 15026978

[jane70290-bib-0072] Schmitz, O. J. , Krivan, V. , & Ovadia, O. (2004). Trophic cascades: The primacy of trait‐mediated indirect interactions. Ecology Letters, 7(2), 153–163. 10.1111/j.1461-0248.2003.00560.x

[jane70290-bib-0073] Schmitz, O. J. , & Suttle, K. B. (2001). Effects of top predator species on direct and indirect interactions in a food web. Ecology, 82(7), 2072–2081. 10.1890/0012-9658(2001)082[2072:EOTPSO]2.0.CO;2

[jane70290-bib-0074] Schroeder, L. A. , & Callaghan, W. M. (1981). Thermal tolerance and acclimation of two species of *Hydra* 1. Limnology and Oceanography, 26(4), 690–696. 10.4319/lo.1981.26.4.0690

[jane70290-bib-0075] Sentis, A. , Hemptinne, J.‐L. , & Brodeur, J. (2012). Using functional response modeling to investigate the effect of temperature on predator feeding rate and energetic efficiency. Oecologia, 169(4), 1117–1125. 10.1007/s00442-012-2255-6 22271203

[jane70290-bib-0076] Shanafelt, D. W. , & Loreau, M. (2018). Stability trophic cascades in food chains. Royal Society Open Science, 5(11), 180995. 10.1098/rsos.180995 30564399 PMC6281913

[jane70290-bib-0077] Shurin, J. B. , Borer, E. T. , Seabloom, E. W. , Anderson, K. , Blanchette, C. A. , Broitman, B. , Cooper, S. D. , & Halpern, B. S. (2002). A cross‐ecosystem comparison of the strength of trophic cascades: Strength of cascades. Ecology Letters, 5(6), 785–791. 10.1046/j.1461-0248.2002.00381.x

[jane70290-bib-0078] Shurin, J. B. , & Seabloom, E. W. (2005). The strength of trophic cascades across ecosystems: Predictions from allometry and energetics. Journal of Animal Ecology, 74(6), 1029–1038. 10.1111/j.1365-2656.2005.00999.x

[jane70290-bib-0079] Singh, S. P. , & Singh, P. (2015). Effect of temperature and light on the growth of algae species: A review. Renewable and Sustainable Energy Reviews, 50, 431–444. 10.1016/j.rser.2015.05.024

[jane70290-bib-0080] Spitze, K. (1991). *Chaoborus* predation and life‐history evolution in *Daphnia pulex*: Temporal pattern of population diversity, fitness, and mean life history. Evolution, 45(1), 82–92. 10.1111/j.1558-5646.1991.tb05268.x 28564082

[jane70290-bib-0081] Stan Development Team . (2024). RStan: The R interface to Stan . (Version 2.32.6) [Computer software]. https://mc‐stan.org/.

[jane70290-bib-0082] Start, D. , & Gilbert, B. (2017). Predator personality structures prey communities and trophic cascades. Ecology Letters, 20(3), 366–374. 10.1111/ele.12735 28120366

[jane70290-bib-0083] Stevenson, C. F. , Demes, K. W. , & Salomon, A. K. (2016). Accounting for size‐specific predation improves our ability to predict the strength of a trophic cascade. Ecology and Evolution, 6(4), 1041–1053. 10.1002/ece3.1870 26941943 PMC4761761

[jane70290-bib-0084] Stockseth, L. , Neale, Z. , & Rudolf, V. H. W. (2025). Strengthening of negative density dependence mediates population decline at high temperatures. Ecology, 106(3), e70030. 10.1002/ecy.70030 40033628

[jane70290-bib-0085] Strickland, M. S. , Hawlena, D. , Reese, A. , Bradford, M. A. , & Schmitz, O. J. (2013). Trophic cascade alters ecosystem carbon exchange. Proceedings of the National Academy of Sciences, 110(27), 11035–11038. 10.1073/pnas.1305191110 PMC3703983 23776213

[jane70290-bib-0086] Su, H. , Feng, Y. , Chen, J. , Chen, J. , Ma, S. , Fang, J. , & Xie, P. (2021). Determinants of trophic cascade strength in freshwater ecosystems: A global analysis. Ecology, 102(7), e03370. 10.1002/ecy.3370 33961286

[jane70290-bib-0087] Terborgh, J. , & Estes, J. A. (2014). Trophic cascades: Predators, prey, and the changing dynamics of nature. Island Press.

[jane70290-bib-0088] Uiterwaal, S. F. , Dell, A. I. , & DeLong, J. P. (2019). Arena size modulates functional responses via behavioral mechanisms. Behavioral Ecology, 30(2), 483–489. 10.1093/beheco/ary188

[jane70290-bib-0089] Uiterwaal, S. F. , & DeLong, J. P. (2020). Functional responses are maximized at intermediate temperatures. Ecology, 101(4), e02975. 10.1002/ecy.2975 31944271

[jane70290-bib-0090] Uszko, W. , Diehl, S. , Englund, G. , & Amarasekare, P. (2017). Effects of warming on predator–prey interactions—A resource‐based approach and a theoretical synthesis. Ecology Letters, 20(4), 513–523. 10.1111/ele.12755 28266168

[jane70290-bib-0091] Walsh, M. R. , & Reznick, D. N. (2008). Interactions between the direct and indirect effects of predators determine life history evolution in a killifish. Proceedings of the National Academy of Sciences, 105(2), 594–599. 10.1073/pnas.0710051105 PMC2206581 18180455

[jane70290-bib-0092] West, D. C. , & Post, D. M. (2016). Impacts of warming revealed by linking resource growth rates with consumer functional responses. Journal of Animal Ecology, 85(3), 671–680. 10.1111/1365-2656.12491 26781835

[jane70290-bib-0093] Wilmers, C. C. , Estes, J. A. , Edwards, M. , Laidre, K. L. , & Konar, B. (2012). Do trophic cascades affect the storage and flux of atmospheric carbon? An analysis of sea otters and kelp forests. Frontiers in Ecology and the Environment, 10(8), 409–415. 10.1890/110176

[jane70290-bib-0094] Wojtal‐Frankiewicz, A. (2012). The effects of global warming on *Daphnia* spp. population dynamics: A review. Aquatic Ecology, 46(1), 37–53. 10.1007/s10452-011-9380-x

[jane70290-bib-0095] Wootton, J. T. (1994a). Predicting direct and indirect effects: An integrated approach using experiments and path analysis. Ecology, 75(1), 151–165. 10.2307/1939391

[jane70290-bib-0096] Wootton, J. T. (1994b). The nature and consequences of indirect effects in ecological communities. Annual Review of Ecology and Systematics, 25, 443–466.

[jane70290-bib-0097] Yan, C. , Stenseth, N. C. , Krebs, C. J. , & Zhang, Z. (2013). Linking climate change to population cycles of hares and lynx. Global Change Biology, 19(11), 3263–3271. 10.1111/gcb.12321 23846828

[jane70290-bib-0098] Zhang, C. , Zhou, Y. , Špoljar, M. , Fressl, J. , Tomljanović, T. , Rama, V. , & Kuczyńska‐Kippen, N. (2023). How can top‐down and bottom‐up manipulation be used to mitigate eutrophication? Mesocosm experiment driven modeling zooplankton seasonal dynamic approach in the trophic cascade. Water Research, 243, 120364. 10.1016/j.watres.2023.120364 37473510

[jane70290-bib-0099] Zhou, L. , Luo, M. , Hong, P. , Leroux, S. , Chen, F. , & Wang, S. (2025). Energy transfer efficiency rather than productivity determines the strength of aquatic trophic cascades. Ecology, 106(1), e4482. 10.1002/ecy.4482 39604056

