# Supplementary material for: Warming increases trophic cascade strength in an aquatic food chain
Source: J Anim Ecol. 2026 Jun 2;95(7):1125–37. doi: 10.1111/1365-2656.70290 (PMC13322182; doi:10.1111/1365-2656.70290)
Supplement: Supplementary file 1 — Table A1. Upper and lower bounds for estimated parameters used in the ODE model fitted to time series data in the absence of Hydra. Table A2. Upper and lower bounds for estimated parameters used in the ODE model fitted to time series data in the presence of Hydra. Table B1. Parameter values μandσ for prior distributions used in the ODE model fitted to time series data at 14°C in the absence of Hydra. Table B2. Parameter values μandσ for prior distributions used in the ODE model fitted to time series data at 17°C in the absence of Hydra. Table B3. Parameter values μandσ for prior distributions used in the ODE model fitted to time series data at 20°C in the absence of Hydra. Table B4. Parameter values μandσ for prior distributions used in the ODE model fitted to time series data at 23°C in the absence of Hydra. Table B5. Parameter values μandσ for prior distributions used in the ODE model fitted to time series data at 26°C in the absence of Hydra. Table B6. Parameter values μandσ for prior distributions used in the ODE model fitted to time series data at 14°C in the presence of Hydra. Table B7. Parameter values μandσ for prior distributions used in the ODE model fitted to time series data at 14°C in the presence of Hydra. Table B8. Parameter values μandσ for prior distributions used in the ODE model fitted to time series data at 20°C in the presence of Hydra. Table B9. Parameter values μandσ for prior distributions used in the ODE model fitted to time series data at 23°C in the presence of Hydra. Table B10. Parameter values μandσ for prior distributions used in the ODE model fitted to time series data at 26°C in the presence of Hydra. Table C1. Summary statistics for 14°C, two species time series data. Gelman‐Rubin statistics R^<1.01 for all parameters verify convergence, neff is the number independent samples. Table C2. Summary statistics for 17°C, two species time series data. Gelman‐Rubin statistics R^<1.01 for all parameters verify convergence, neff is the number [file JANE-95-1125-s001.docx]

Supplementary information: Warming increases the strength of trophic cascades.

**Table of Contents:**

S1:Statistical model ………………………………………………………………………….. 2 - 7

S1A: *Likelihood statement*……………………………………………………………….. 2

S1B: *Prior Distributions*…………………………………………………………………. 3

S2: Fit Output Summary ………………………………………………………………………… 9

S3: Fit Trace Plots ……………………………………………………………………………… 19

S4: Change in Average Trophic Cascade Strength …………………………………………….. 29

S5: Supplementary Plots From Text …………………………………………………………… 30

**S1: Statistical Model:**

*S1A :Likelihood statement:*

In the main text, a major goal of our experiment was to statistically fit ordinary differential equation models (ODEs) to the dynamics of the two- and three-species food chains across different temperatures to provide mechanistic insights into why the population dynamics differed across temperatures and treatments. To do so, we used a trajectory matching framework and fit the ODE models using a Bayesian approach. Here we describe our approach and the exact model we used to fit the ODE’s to the population dynamics data.

Using the Bayesian approach, our goal was to estimate the posterior distributions of the ODE parameters $\theta$using the data $y$ ($p(\theta|y)$) by combining the likelihood ($p(y|\theta)$) and priors on the parameters ($p(\theta)$). The approach we used to do so is known as ‘trajectory matching’ which assumes that the observed error in the data around the ODE solutions is purely observational error. Specifically, for each iteration of the Hamiltonian Monte Carlo sampling used to approximate the posterior distribution, we use the sampled parameters $\theta$ along with estimated initial values $N_{i,0}$ ,where i denotes the species (Predators (P), Consumers (C), and Resources (R)) and 0 denotes the initial time point, to get a numerical solution of the appropriate set of ODEs across times t for each species i, $N_{i,t}$ (see equations 1 and 2 in the main text). Once we have this numerical solution of the ODE, we can then calculate a likelihood of observed data $Y_{i,t}$where again i denotes the species and t denotes the time point. Specifically, we modeled the likelihood for the daphnia and algae equations as

$$Y_{i,t}\sim\mathcal{N}\left( N_{i,t},\sigma_{i}^{2} \right),\quad i=C,R;\quad j=1,\ldots,t$$

Where $\mathcal{N}$ denotes a normal distribution with the mean given by the numerical solution of the ODE at time t with species-specific observation error given by the variance $\sigma_{i}^{2}$. By coupling this likelihood for each species with prior probability distributions for the ODE and statistical model parameters $\theta$ and the initial values $N_{i,0}$, we were able to approximate the posterior distributions of the parameters given the data.

For hydra equations, we used a similar approach to model the likelihood, but used a lognormal distribution:

$$Y_{i,t}\mathcal{\sim L}\mathcal{N}\left( log(N_{i,t}+1\times{10}^{-6}),\tau_{i}^{2} \right),\quad i=P;\quad j=1,\ldots,t$$

Where $\mathcal{LN}$ denotes a lognormal distribution with the mean given by the log of the numerical solution of the ODE at time t with species-specific observation error given by the variance $\tau_{i}^{2}$. We did this to allow for variance to scale with abundance, consistent with demographic or environmental stochasticity, and avoid the assumption of constant variance. Note that $\tau_{i}^{2}$ is the standard deviation of the log of the distribution. Furthermore, we add 1x10^-6^ to *N_i,t_* to buffer against a solution where *N_i,t_* = log(0).

To implement this model with our data, we used a mostly ‘complete pooling’ approach in which we fit the model for each temperature-treatment combination across all replicates simultaneously. The only parameters that we allowed to vary across the replicates were the initial values. Thus, we assumed that the parameters governing the population dynamics in each replicate were identical but that replicates could differ in their initial values. To complete our description of the statistical model, we give the priors that we used below. All code for fitting the models is also available (see Data Availability Statement of the main text).

*S1B: Prior Distributions:*

Initial values for the ODE state variables at time *t = 0* are estimated using the following priors:

$$N_{R,0,m}\sim\mathcal{N}\left( Y_{R,1,m},\sigma=1.5\times{10}^{4} \right)$$

$$N_{C,0,m}\sim\mathcal{N}\left( Y_{C,1,m},\sigma=0.2 \right)$$

$$N_{P,0,m}\sim\mathcal{N}\left( Y_{P,1,m},\sigma=0.1 \right)$$

Where $N_{i,0,m}$ is the estimated initial abundance of species *i* in replicate *m* and $Y_{i,1,m}$, is the first observed value in the time series for the same species *i* and replicate *m*.

Observation errors are given the following priors:

$$\sigma_{R}^{2}\sim\text{Exponential}\left( 1\times{10}^{5} \right)$$

$$\sigma_{C}^{2}\sim\text{Exponential}\left( 1 \right)$$

$${log(\tau}_{P}^{2})\sim\text{Exponential}\left( 1 \right)$$

Where $\sigma_{i}^{2}$ is the estimated observation error for species *R* and *C* that is exponentially distributed and $\tau_{i}^{2}$is the estimated observation error for species *P* that is exponentially distributed. We estimate the observation error of hydra (*P*) on the log scale since the likelihood for the hydra equation used a lognormal distribution

ODE specific parameters $\mathcal{X}$ are modeled with truncated-normal priors:

$$\mathcal{X\sim}\mathcal{N}\left( \mu,\sigma\right)\mathcal{T[}upper, lower],\quad\mathcal{X}=\{r,Q,a_{c},e_{c},d_{c},h_{c},w_{c},B,a_{p},e_{p},d_{p},h_{p},w_{p}\}$$

Where $\mu$ and $\sigma$ denote the mean and standard deviation of the prior distributions. The priors are normally distributed and truncated with lower bounds and upper bounds. We used truncated-normal distributions to prevent the solver from entering parameter space that is not biologically relevant. See tables A1 & A2 for treatment specific parameter bounds. Since time series data were fit separately for each treatment combination, prior values for parameters may vary between treatments. See Supplementary Tables B1–B10 for treatment-specific prior specifications.

**Table A1.** Upper and lower bounds for estimated parameters used in the ODE model fitted to time series data in the absence of *Hydra.*

| Number of trophic levels - 2 | | |
| --- | --- | --- |
| Parameter $\left( \theta\right)$ | Lower Bound | Upper Bound |
| *r* | $0$ | $2$ |
| *Q* | $0$ | $1\times{10}^{-3}$ |
| $a_{c}$ | $0$ | 2 |
| $e_{c}$ | $0$ | 0.01 |
| $d_{c}$ | 0 | NA |
| $h_{c}$ | $0$ | $1\times{10}^{-3}$ |
| $w_{c}$ | 0 | NA |
| *B* | $0$ | $0.4$ |

**Table A2.** Upper and lower bounds for estimated parameters used in the ODE model fitted to time series data in the presence of *Hydra.*

| Number of trophic levels - 3 | | |
| --- | --- | --- |
| Parameter $\left( \theta\right)$ | Lower Bound | Upper Bound |
| *r* | $0$ | $2$ |
| *Q* | $0$ | $1\times{10}^{-3}$ |
| $a_{c}$ | $0$ | NA |
| $h_{c}$ | $0$ | 0.001 |
| $w_{c}$ | 0 | NA |
| $a_{p}$ | $0$ | NA |
| $e_{p}$ | 0 | 1 |
| $d_{p}$ | $0$ | 1 |
| $h_{p}$ | 0 | 1 |
| $w_{p}$ | 0 | NA |

**Table B1.** Parameter values $(\mu and \sigma)$ for prior distributions used in the ODE model fitted to time series data at 14°C in the absence of *Hydra.*

| Temperature – 14, Number of trophic levels - 2 | | |
| --- | --- | --- |
| Parameter $\left( \theta\right)$ | $\mu$ | $\sigma$ |
| *r* | $0.05$ | $9.2\times{10}^{-3}$ |
| *Q* | $3.5\times{10}^{-7}$ | $2.7\times{10}^{-7}$ |
| $a_{c}$ | $0.2$ | $0.12$ |
| $e_{c}$ | $5.6\times{10}^{-4}$ | $2.9\times{10}^{-4}$ |
| $d_{c}$ | 2.4 | $0.65$ |
| $h_{c}$ | $1.9\times{10}^{-4}$ | $9.6\times{10}^{-5}$ |
| $w_{c}$ | 2.5 | 0.35 |
| *B* | $0.02$ | $0.02$ |

**Table B2.** Parameter values $(\mu and \sigma)$ for prior distributions used in the ODE model fitted to time series data at 17°C in the absence of *Hydra.*

| Temperature – 17, Number of trophic levels - 2 | | |
| --- | --- | --- |
| Parameter $\left( \theta\right)$ | $\mu$ | $\sigma$ |
| *r* | $0.09$ | 0.01 |
| *Q* | $8.4\times{10}^{-8}$ | $6.2\times{10}^{-8}$ |
| $a_{c}$ | 0.2 | 0.1 |
| $e_{c}$ | $2.2\times{10}^{-5}$ | $6.9\times{10}^{-6}$ |
| $d_{c}$ | 1 | 0.3 |
| $h_{c}$ | $4.2\times{10}^{-6}$ | $3\times{10}^{-6}$ |
| $w_{c}$ | 1.9 | 0.39 |
| *B* | 0.08 | 0.03 |

**Table B3.** Parameter values $(\mu and \sigma)$ for prior distributions used in the ODE model fitted to time series data at 20°C in the absence of *Hydra.*

| Temperature – 20, Number of trophic levels - 2 | | |
| --- | --- | --- |
| Parameter $\left( \theta\right)$ | $\mu$ | $\sigma$ |
| *r* | 0.06 | 1 |
| *Q* | 1.0e-7 | 1.0e-7 |
| $a_{c}$ | 0.2 | 1 |
| $e_{c}$ | 0.001 | 1 |
| $d_{c}$ | 0.2 | 1 |
| $h_{c}$ | 1e-8 | 1 |
| $w_{c}$ | 1 | 1 |
| *B* | 0.1 | 0.05 |

**Table B4.** Parameter values $(\mu and \sigma)$ for prior distributions used in the ODE model fitted to time series data at 23°C in the absence of *Hydra.*

| Temperature – 23, Number of trophic levels - 2 | | |
| --- | --- | --- |
| Parameter $\left( \theta\right)$ | $\mu$ | $\sigma$ |
| *r* | 0.06 | 1 |
| *Q* | 1.0e-7 | 1.0e-7 |
| $a_{c}$ | 0.2 | 1 |
| $e_{c}$ | 0.001 | 1 |
| $d_{c}$ | 0.2 | 1 |
| $h_{c}$ | 1e-8 | 1 |
| $w_{c}$ | 1 | 1 |
| *B* | 0.1 | 0.05 |

**Table B5.** Parameter values $(\mu and \sigma)$ for prior distributions used in the ODE model fitted to time series data at 26°C in the absence of *Hydra.*

| Temperature – 26, Number of trophic levels - 2 | | |
| --- | --- | --- |
| Parameter $\left( \theta\right)$ | $\mu$ | $\sigma$ |
| *r* | 0.06 | 1 |
| *Q* | 1.0e-7 | 1.0e-7 |
| $a_{c}$ | 0.2 | 1 |
| $e_{c}$ | 0.001 | 1 |
| $d_{c}$ | 0.2 | 1 |
| $h_{c}$ | 1e-8 | 1 |
| $w_{c}$ | 1 | 1 |
| *B* | 0.1 | 0.05 |

**Table B6.** Parameter values $(\mu and \sigma)$ for prior distributions used in the ODE model fitted to time series data at 14°C in the presence of *Hydra.*

| Temperature – 14, Number of trophic levels - 3 | | |
| --- | --- | --- |
| Parameter $\left( \theta\right)$ | $\mu$ | $\sigma$ |
| *r* | 0.05 | $5.4\times{10}^{-3}$ |
| *Q* | $3.3\times{10}^{-7}$ | $1.7\times{10}^{-7}$ |
| $a_{c}$ | 7.5 | 2.3 |
| $h_{c}$ | $1.9\times{10}^{-4}$ | $6.4\times{10}^{-5}$ |
| $w_{c}$ | 2.5 | 0.3 |
| $a_{p}$ | 16.8 | 4 |
| $e_{p}$ | 0.07 | 0.01 |
| $d_{p}$ | 0.05 | $7.2\times{10}^{-3}$ |
| $h_{p}$ | 0.2 | 0.04 |
| $w_{p}$ | 22.4 | 7.9 |

**Table B7.** Parameter values $(\mu and \sigma)$ for prior distributions used in the ODE model fitted to time series data at 14°C in the presence of *Hydra.*

| Temperature – 17, Number of trophic levels - 3 | | |
| --- | --- | --- |
| Parameter $\left( \theta\right)$ | $\mu$ | $\sigma$ |
| *r* | 0.09 | $7.9\times{10}^{-3}$ |
| *Q* | $7.9\times{10}^{-8}$ | $4.2\times{10}^{-8}$ |
| $a_{c}$ | 5 | 3 |
| $h_{c}$ | $4.4\times{10}^{-6}$ | $2.2\times{10}^{-6}$ |
| $w_{c}$ | 2 | 0.3 |
| $a_{p}$ | 18.6 | 4 |
| $e_{p}$ | 0.1 | 0.02 |
| $d_{p}$ | 0.03 | $7.7\times{10}^{-3}$ |
| $h_{p}$ | 0.2 | 0.05 |
| $w_{p}$ | 31 | 8 |

**Table B8.** Parameter values $(\mu and \sigma)$ for prior distributions used in the ODE model fitted to time series data at 20°C in the presence of *Hydra.*

| Temperature – 20, Number of trophic levels - 3 | | |
| --- | --- | --- |
| Parameter $\left( \theta\right)$ | $\mu$ | $\sigma$ |
| *r* | 0.1 | 0.02 |
| *Q* | $1.8\times{10}^{-7}$ | $8.5\times{10}^{-8}$ |
| $a_{c}$ | 5 | 3 |
| $h_{c}$ | $4.3\times{10}^{-4}$ | $6,2\times{10}^{-4}$ |
| $w_{c}$ | 1 | 0.6 |
| $a_{p}$ | 33.6 | 15 |
| $e_{p}$ | 0.08 | 0.02 |
| $d_{p}$ | 0.04 | 0.01 |
| $h_{p}$ | 0.15 | 0.07 |
| $w_{p}$ | 12 | 11 |

**Table B9.** Parameter values $(\mu and \sigma)$ for prior distributions used in the ODE model fitted to time series data at 23°C in the presence of *Hydra.*

| Temperature – 23, Number of trophic levels - 3 | | |
| --- | --- | --- |
| Parameter $\left( \theta\right)$ | $\mu$ | $\sigma$ |
| *r* | 0.24 | 0.04 |
| *Q* | $1.3\times{10}^{-7}$ | $7.8\times{10}^{-8}$ |
| $a_{c}$ | 5 | 3 |
| $h_{c}$ | $8.9\times{10}^{-7}$ | $7.7\times{10}^{-7}$ |
| $w_{c}$ | 2 | 0.5 |
| $a_{p}$ | 32 | 19 |
| $e_{p}$ | 0.08 | 0.03 |
| $d_{p}$ | 0.03 | $6.5\times{10}^{-3}$ |
| $h_{p}$ | 0.2 | 0.07 |
| $w_{p}$ | 10 | 12 |

**Table B10.** Parameter values $(\mu and \sigma)$ for prior distributions used in the ODE model fitted to time series data at 26°C in the presence of *Hydra.*

| Temperature – 26, Number of trophic levels - 3 | | |
| --- | --- | --- |
| Parameter $\left( \theta\right)$ | $\mu$ | $\sigma$ |
| *r* | 0.2 | 0.05 |
| *Q* | $2.2\times{10}^{-7}$ | $9.0\times{10}^{-8}$ |
| $a_{c}$ | 5 | 3 |
| $h_{c}$ | $1.5\times{10}^{-5}$ | $4.3\times{10}^{-6}$ |
| $w_{c}$ | 1.58 | 0.7 |
| $a_{p}$ | 34 | 15 |
| $e_{p}$ | 0.08 | 0.02 |
| $d_{p}$ | 0.04 | 0.01 |
| $h_{p}$ | 0.15 | 0.07 |
| $w_{p}$ | 12 | 12 |

**S2:Fit Output Summary:**

**Table C1:** Summary statistics for 14°C, two species time series data. Gelman-Rubin statistics $\hat{R}<1.01$ for all parameters verify convergence, $n_{eff}$ is the number independent samples.

**Table C2:** Summary statistics for 17°C, two species time series data. Gelman-Rubin statistics $\hat{R}<1.01$ for all parameters verify convergence, $n_{eff}$ is the number independent samples.

**Table C3:** Summary statistics for 20°C, two species time series data. Gelman-Rubin statistics $\hat{R}<1.01$ for all parameters verify convergence, $n_{eff}$ is the number independent samples.

**Table C4:** Summary statistics for 23°C, two species time series data. Gelman-Rubin statistics $\hat{R}<1.01$ for all parameters verify convergence, $n_{eff}$ is the number independent samples.

**Table C5:** Summary statistics for 26°C, two species time series data. Gelman-Rubin statistics $\hat{R}<1.01$ for all parameters verify convergence, $n_{eff}$ is the number independent samples.

**Table C6:** Summary statistics for 14°C, three species time series data. Gelman-Rubin statistics $\hat{R}<1.01$ for all parameters verify convergence, $n_{eff}$ is the number independent samples.

**Table C7:** Summary statistics for 17°C, three species time series data. Gelman-Rubin statistics $\hat{R}<1.01$ for all parameters verify convergence, $n_{eff}$ is the number independent samples.

**Table C8:** Summary statistics for 20°C, three species time series data. Gelman-Rubin statistics $\hat{R}<1.01$ for all parameters verify convergence, $n_{eff}$ is the number independent samples.

**Table C9:** Summary statistics for 23°C, three species time series data. Gelman-Rubin statistics $\hat{R}<1.01$ for all parameters verify convergence, $n_{eff}$ is the number independent samples.

**Table C10:** Summary statistics for 26°C, three species time series data. Gelman-Rubin statistics $\hat{R}<1.01$ for all parameters verify convergence, $n_{eff}$ is the number independent samples.

**S3: Fit trace plots:**


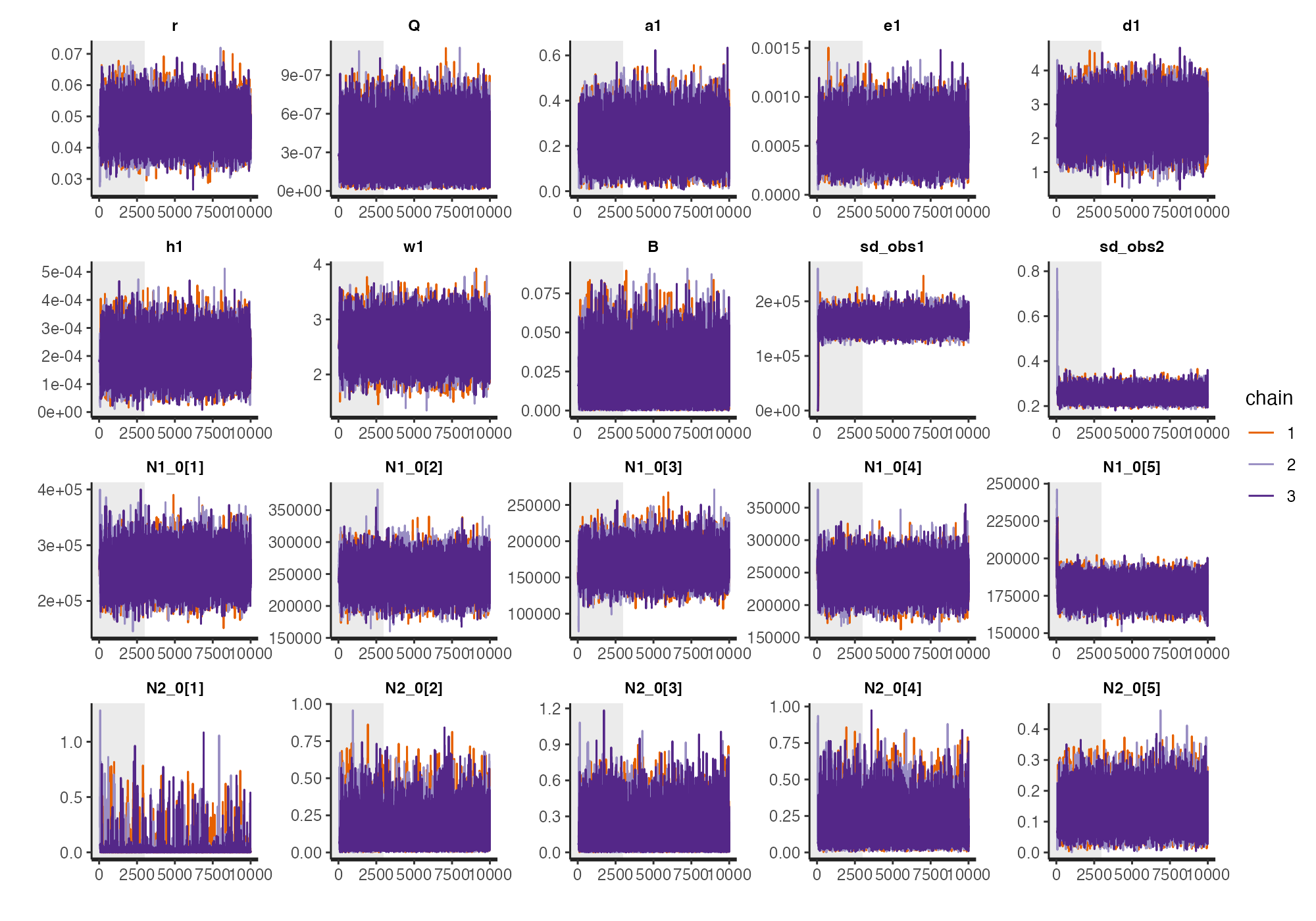


**Figure A1.** Trace plots of MCMC chains for the two-species time series data at 14 °C. Grey shaded areas indicate warm-up iterations.


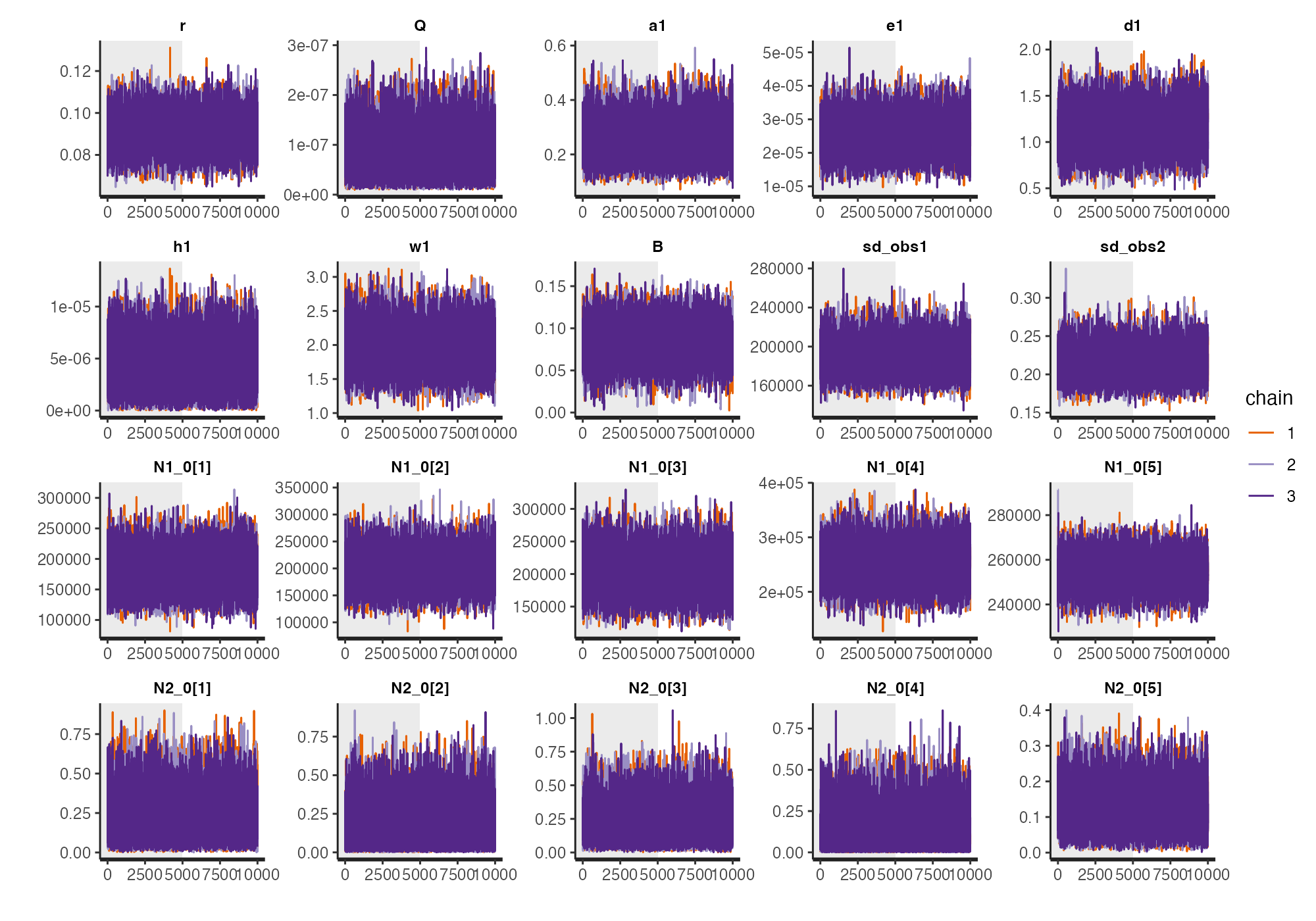


**Figure A2.** Trace plots of MCMC chains for the two-species time series data at 17 °C. Grey shaded areas indicate warm-up iterations.


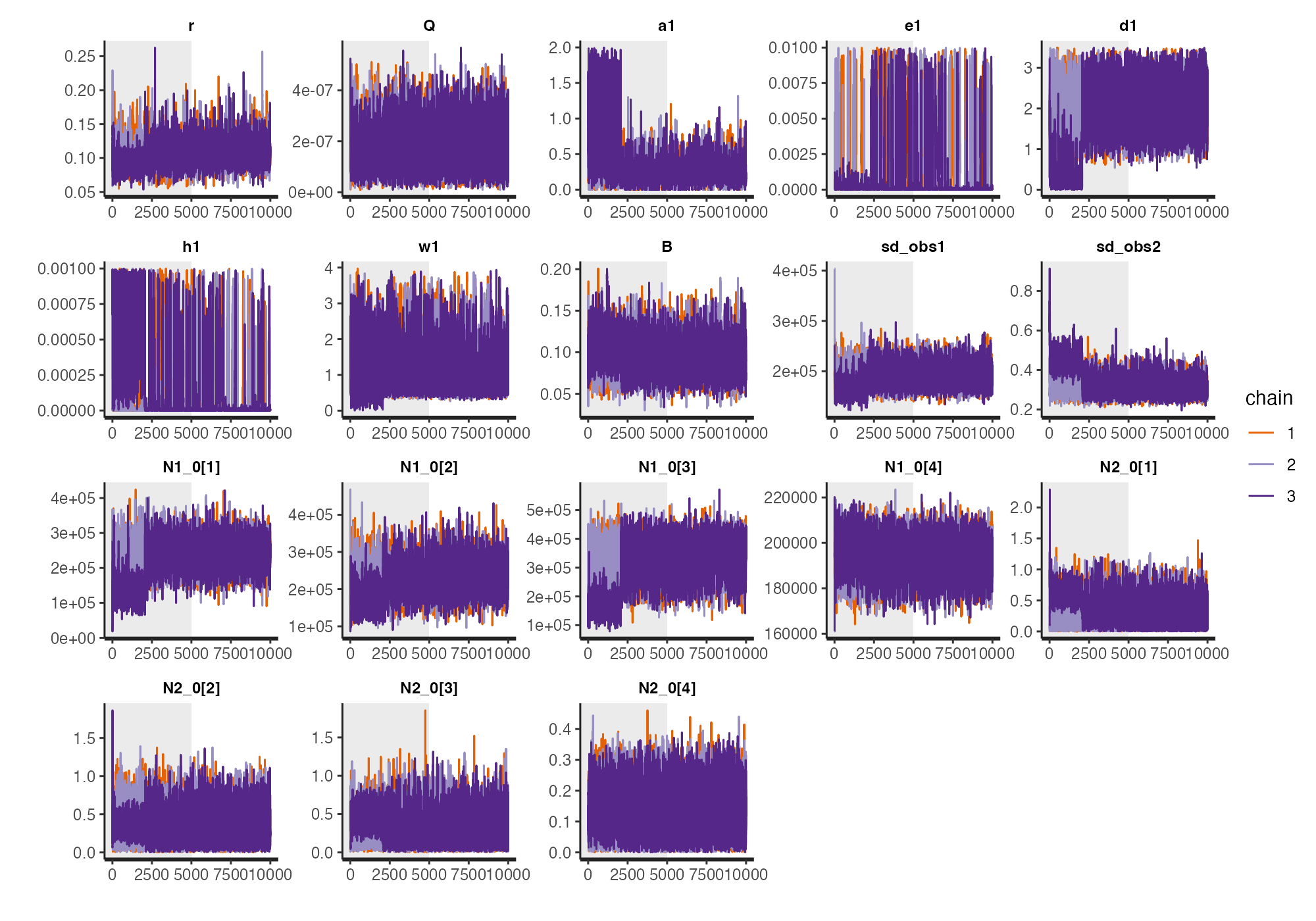


**Figure A3.** Trace plots of MCMC chains for the two-species time series data at 20 °C. Grey shaded areas indicate warm-up iterations.


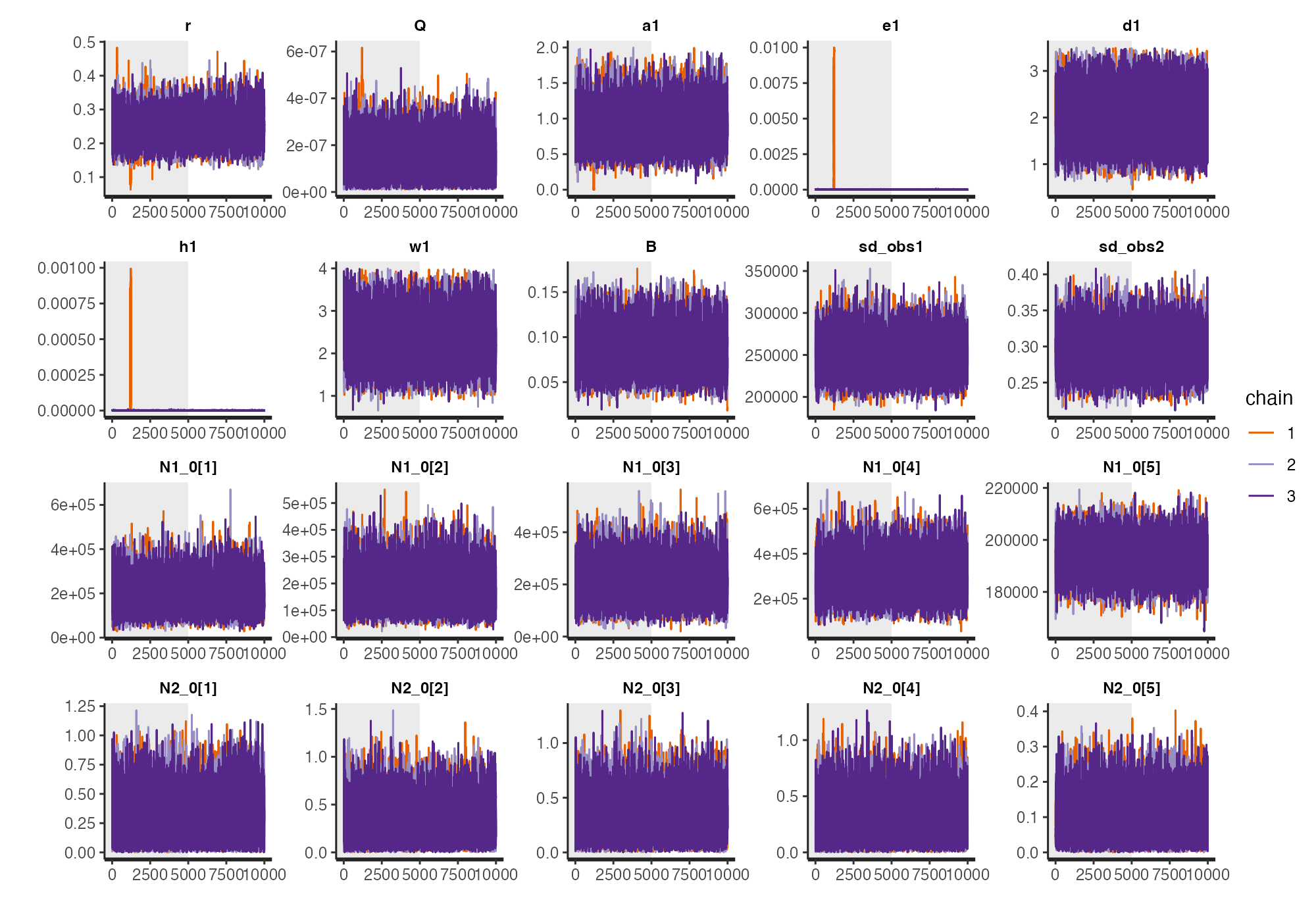


**Figure A4.** Trace plots of MCMC chains for the two-species time series data at 23 °C. Grey shaded areas indicate warm-up iterations.


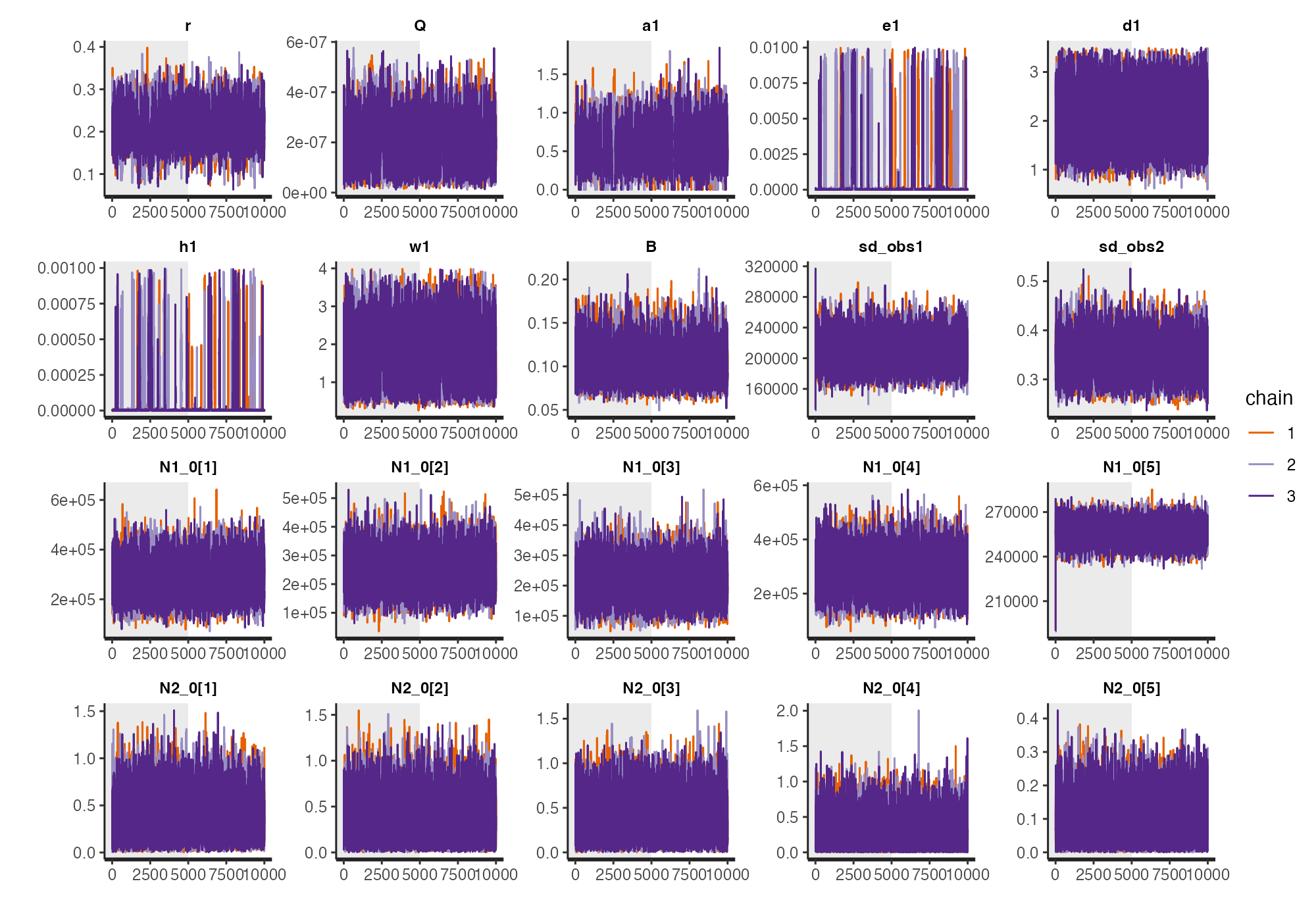


**Figure A5.** Trace plots of MCMC chains for the two-species time series data at 26 °C. Grey shaded areas indicate warm-up iterations.


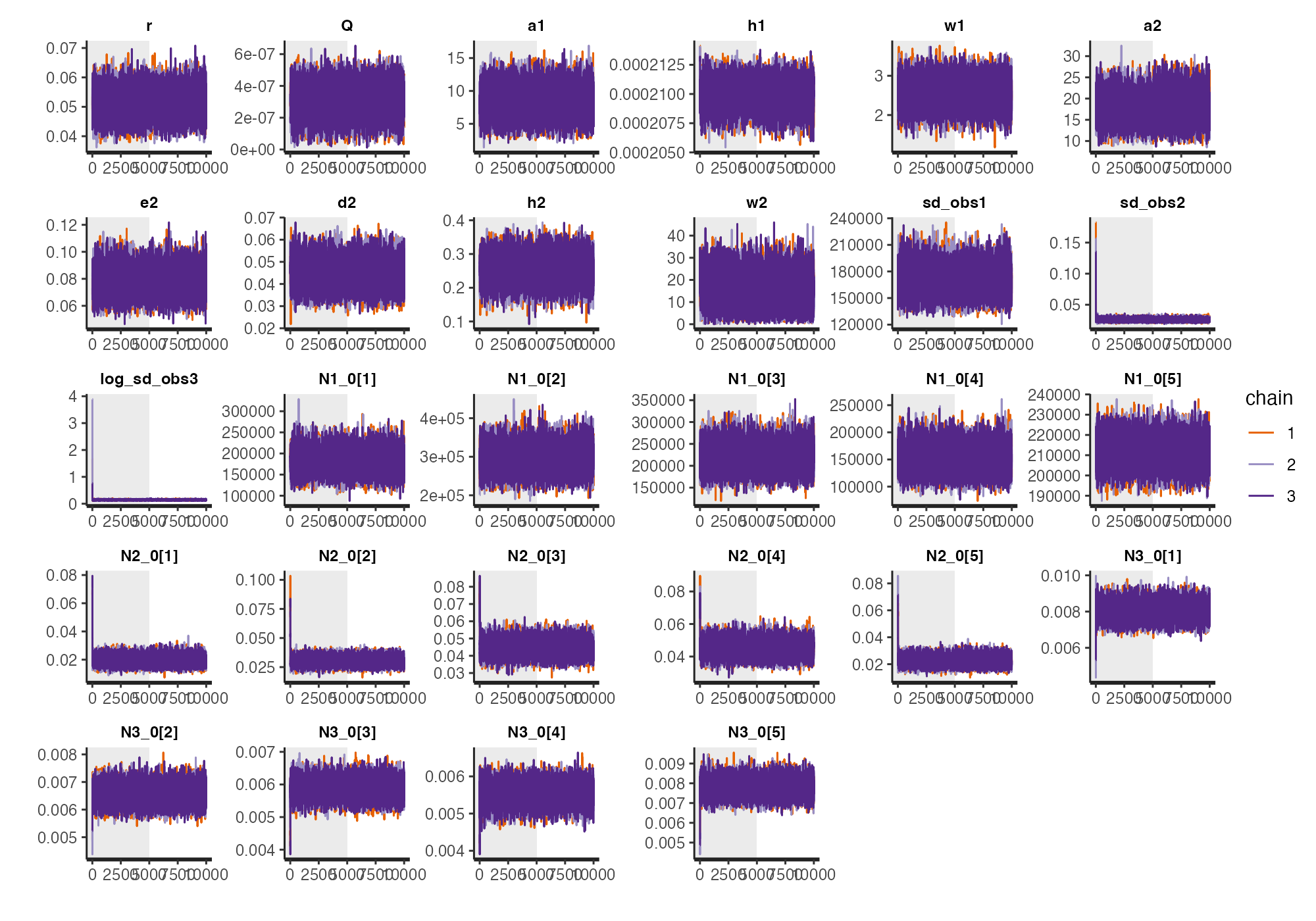


**Figure A6.** Trace plots of MCMC chains for the three-species time series data at 14 °C. Grey shaded areas indicate warm-up iterations.


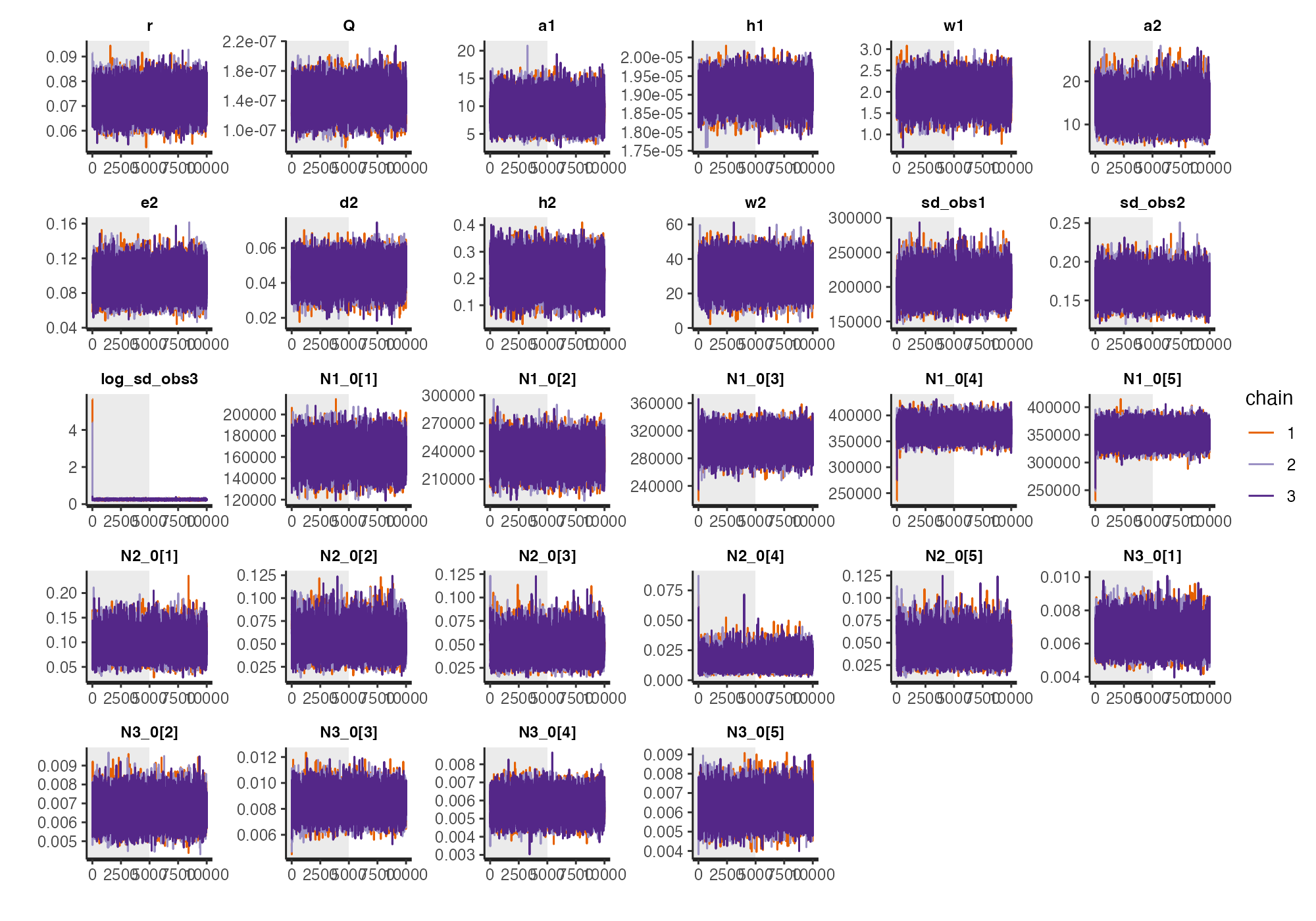


**Figure A7.** Trace plots of MCMC chains for the three-species time series data at 17 °C. Grey shaded areas indicate warm-up iterations.


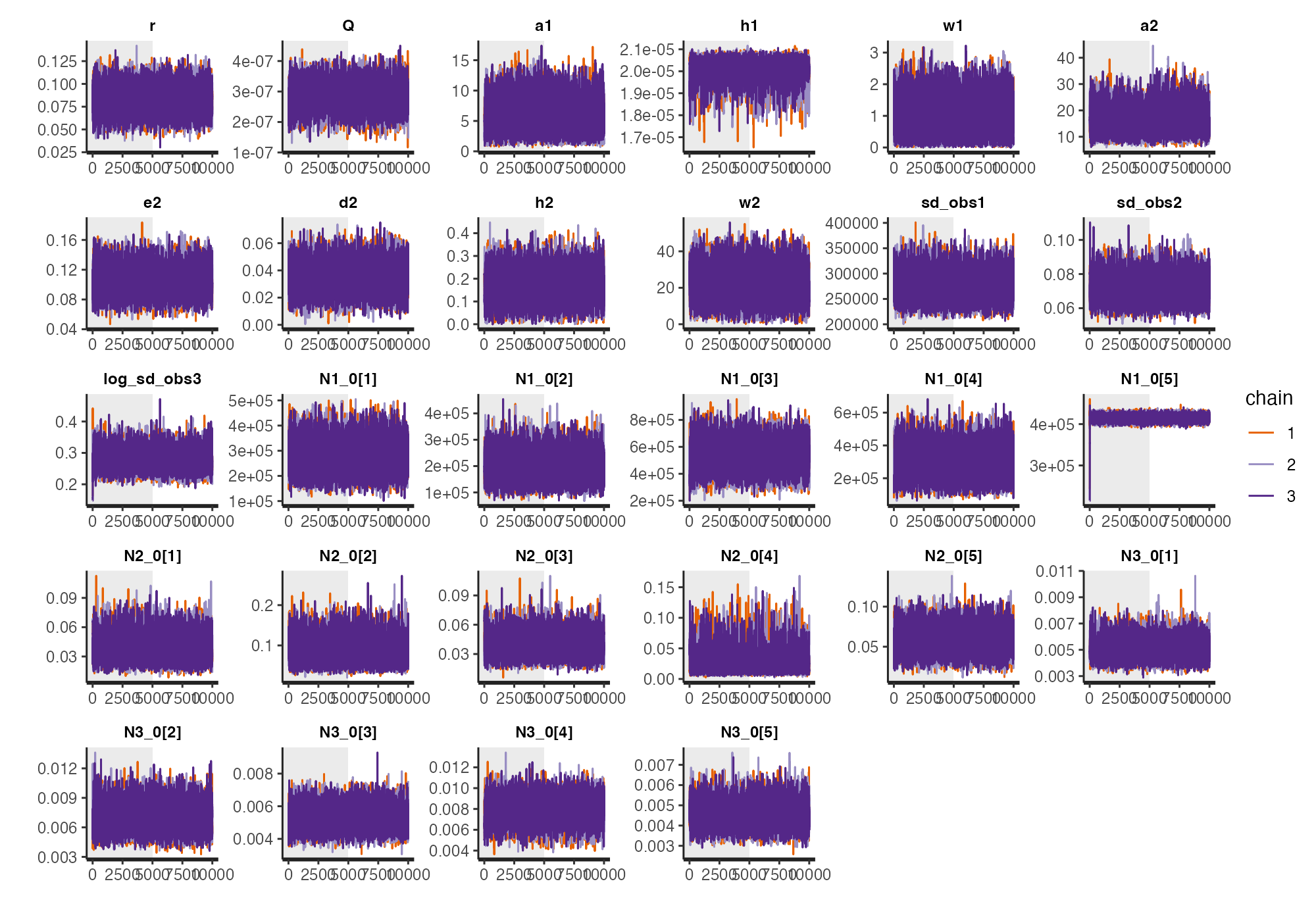


**Figure A8.** Trace plots of MCMC chains for the three-species time series data at 20 °C. Grey shaded areas indicate warm-up iterations.


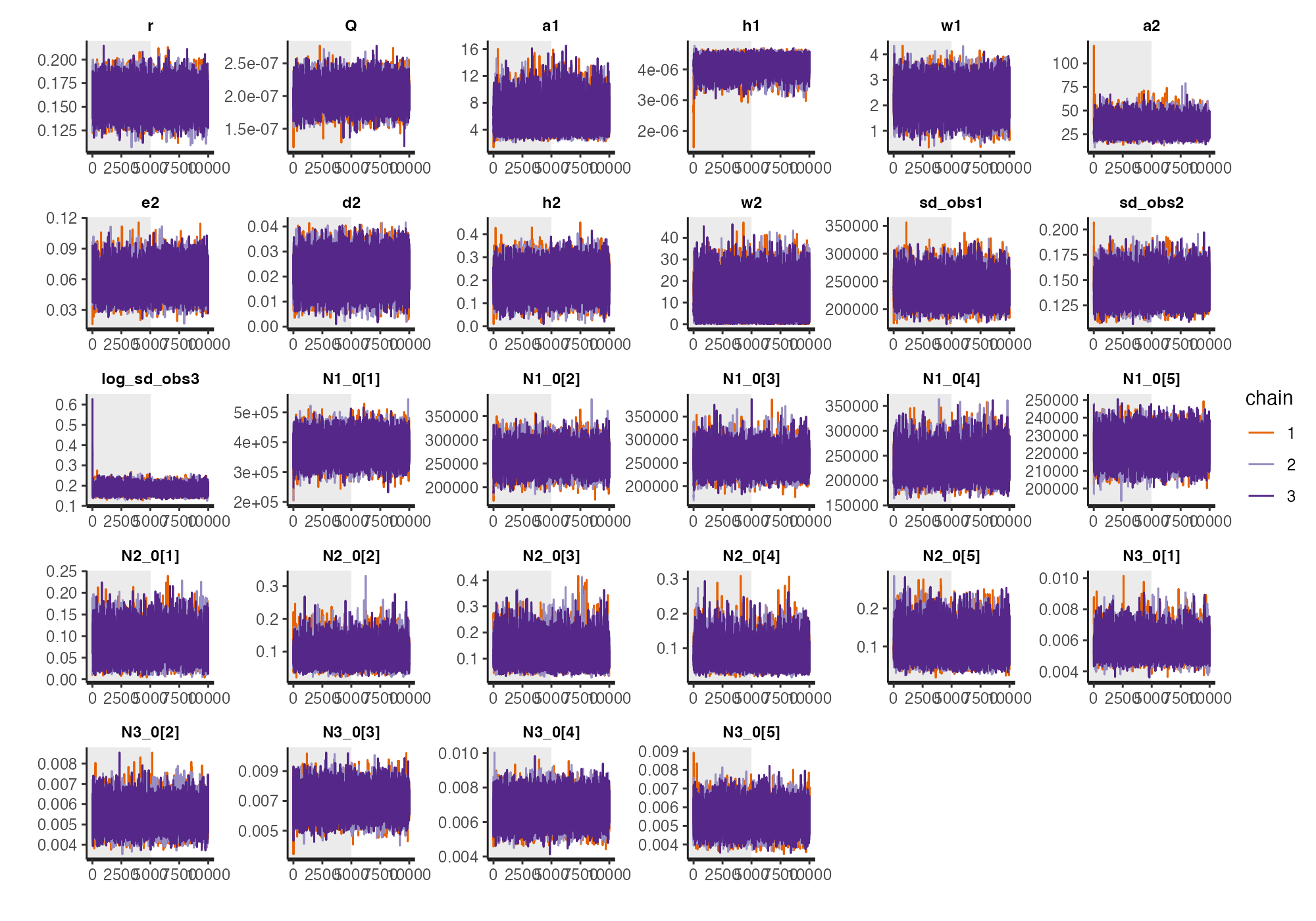


**Figure A9.** Trace plots of MCMC chains for the three-species time series data at 23 °C. Grey shaded areas indicate warm-up iterations.


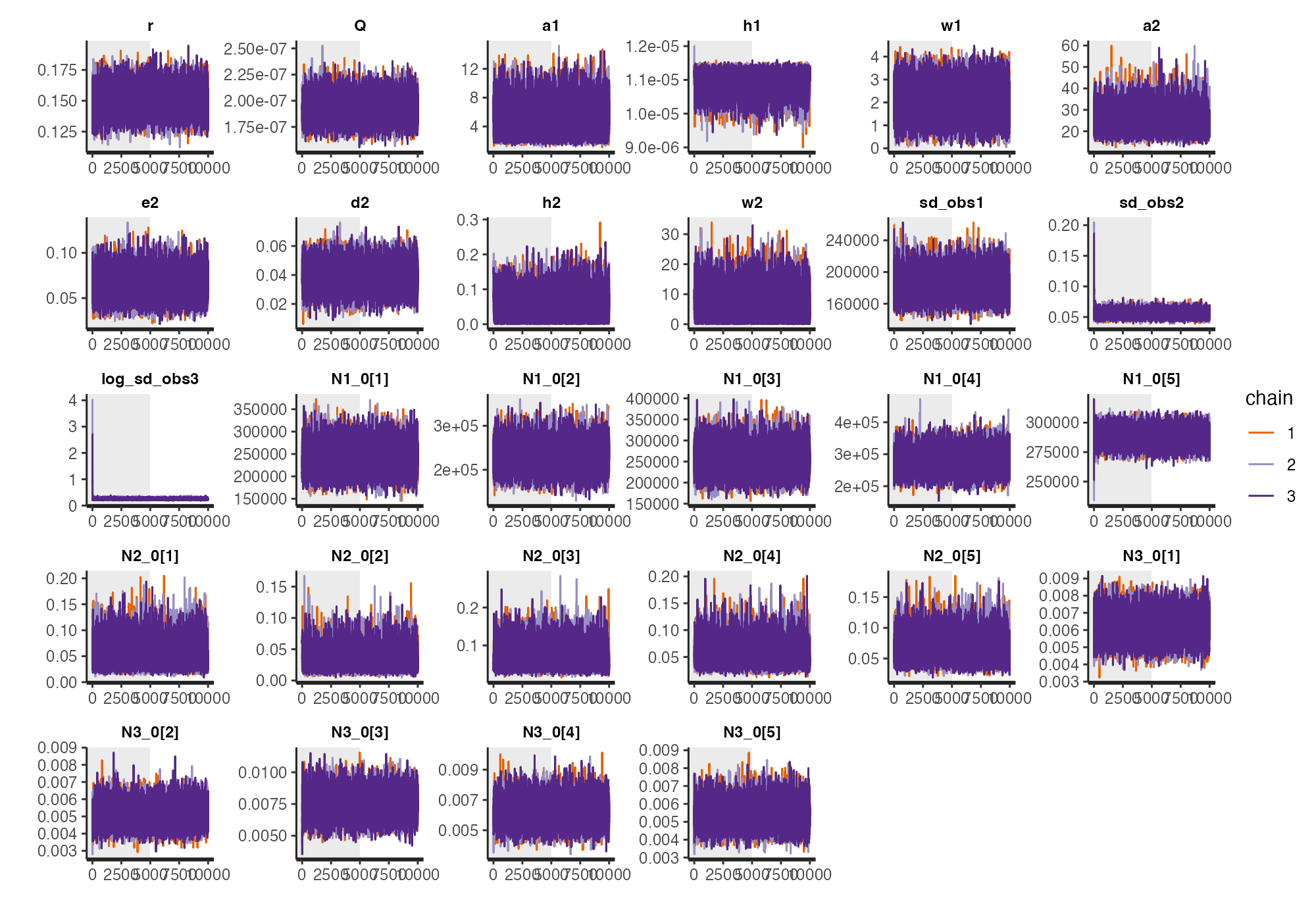


**Figure A10.** Trace plots of MCMC chains for the three-species time series data at 26 °C. Grey shaded areas indicate warm-up iterations.

**S4 :Change in Trophic Cascade Strength**

In the main text, we describe our approach to quantifying how mean trophic cascade strength changes across the temperature gradient (see Methods, Main Text). Here, we provide additional detail. To estimate the relationship between temperature and trophic cascade strength, we fit linear regressions to posterior estimates of average cascade strength. Importantly, we did not pool data to fit a single regression across the posterior distributions. Instead, for each of the 6,000 posterior samples, we extracted one estimate of average cascade strength from each of the five temperature treatments and fit a linear regression to those five points. This process yielded 6,000 slope estimates, forming a posterior distribution of slopes describing how cascade strength changed from 14°C to 26°C (Fig. B1). Of these, 14,792 had a positive slope, indicating a 98.61% posterior probability that mean trophic cascade strength increased with temperature.


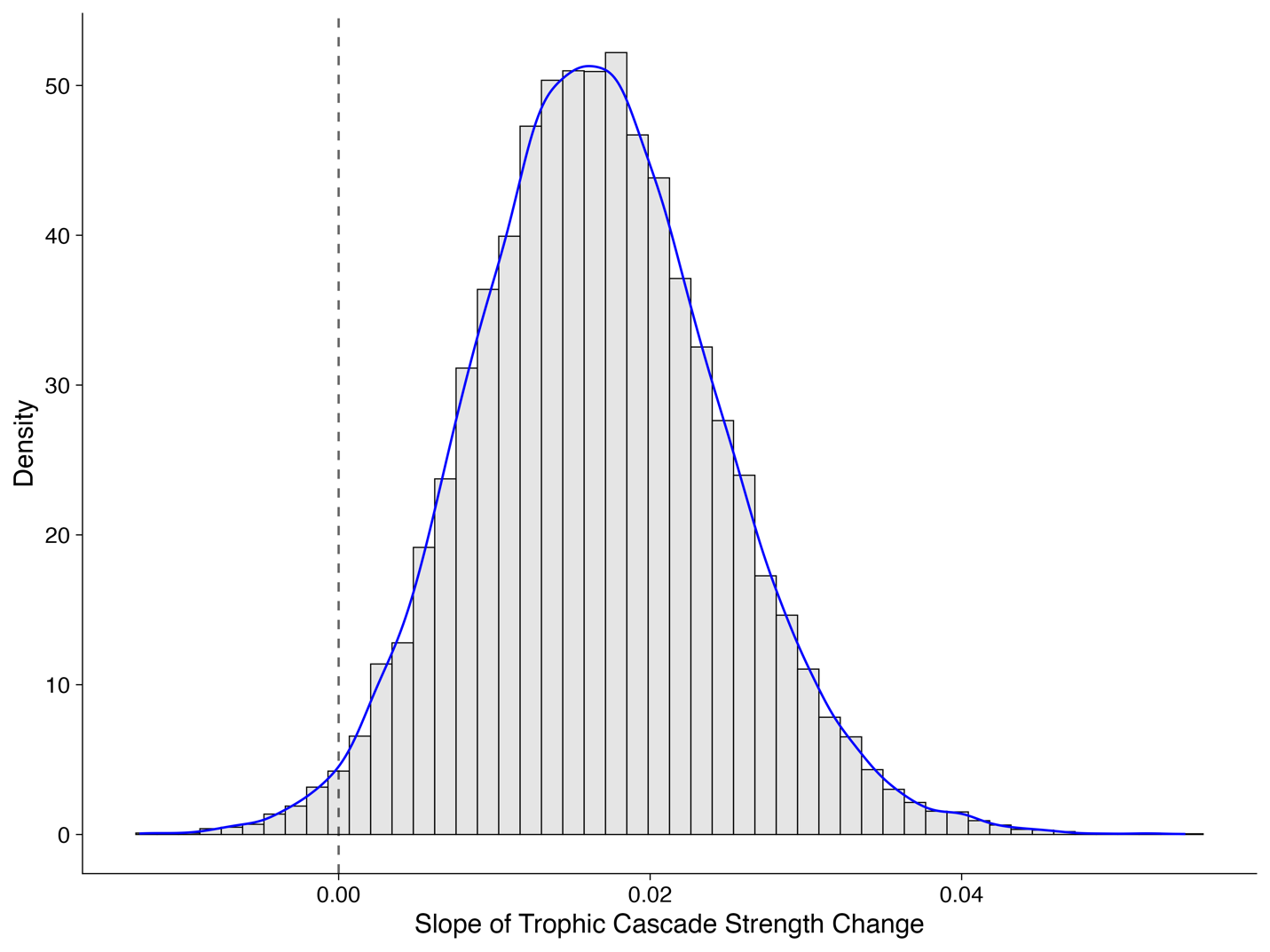


Figure B1. Posterior distribution of slope estimates from linear regressions fit to posterior samples of average trophic cascade strength across temperature treatments. The blue curve shows the density of slope values, and the grey bars represent their frequency. The vertical dashed line indicates a slope of zero. Out of 15,000 regressions, 98.61% yielded positive slopes (N = 15,000).

**S5 :Supplementary Plots From Text**

**
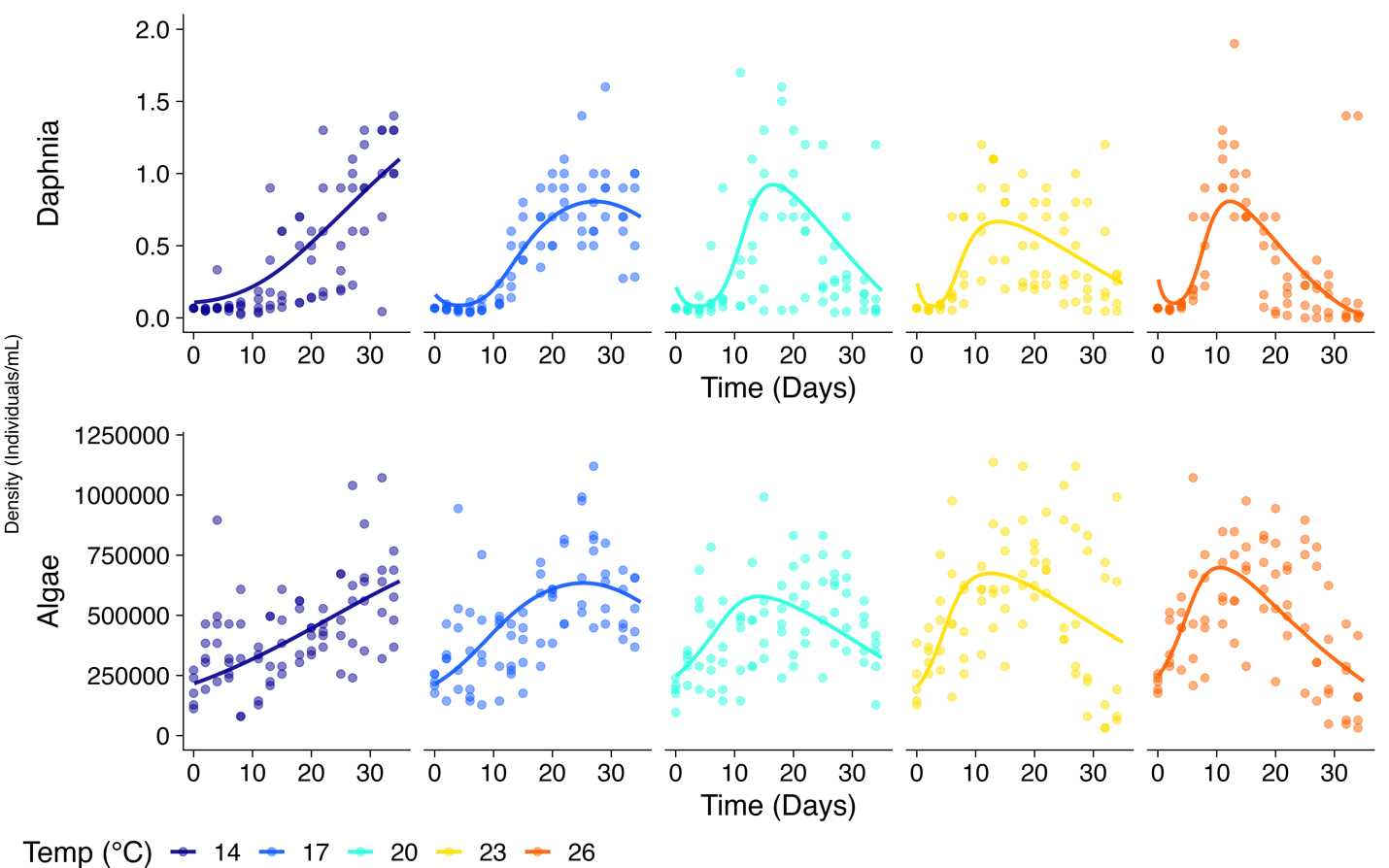
**

Figure C1: **Supplements** **Dynamics of interacting daphnia, and algae populations from two-species fits.** Points are replicate-level population densities on each sampling day. Lines are the posterior predictive mean of ODE solutions calculated from parameter values sampled across posterior distributions (Main Text: Equation 1). Temperatures are shown from cool to warm colors. The top row shows dynamics of daphnia populations in the absence of hydra. The bottom row shows the dynamics of algae populations in the absence of hydra.

**
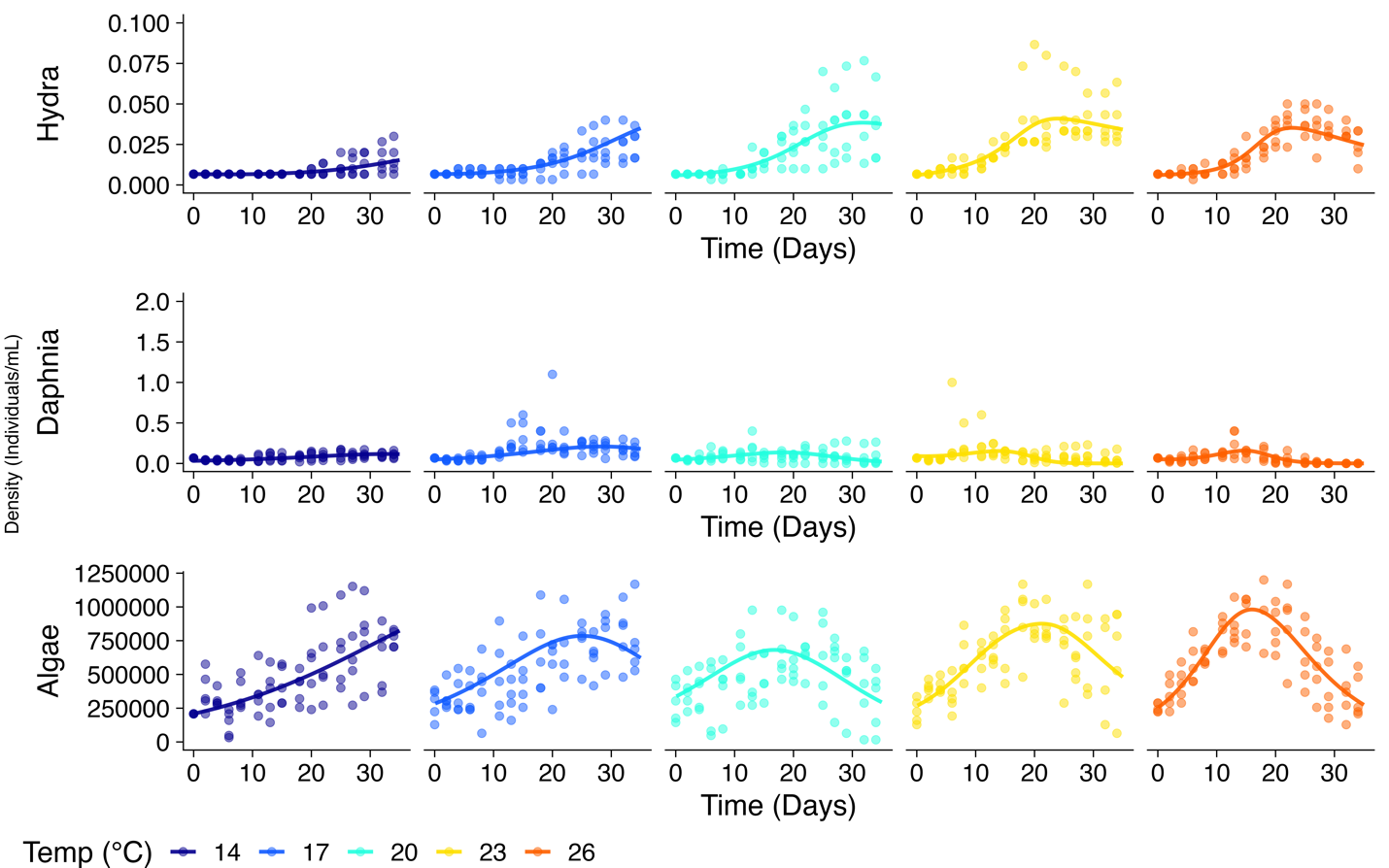
**

Figure C2: **Dynamics of interacting daphnia, and algae populations from three-species fits.** Points are replicate-level population densities on each sampling day. Lines are the posterior predictive mean of ODE solutions calculated from parameter values sampled across posterior distributions (Main Text: Equation 2). Temperatures are shown from cool to warm colors. The top row shows dynamics of hydra populations. The middle row shows the dynamics of daphnia populations in the presence of hydra. The bottom row shows the dynamics of algae populations in the presence of hydra.

**
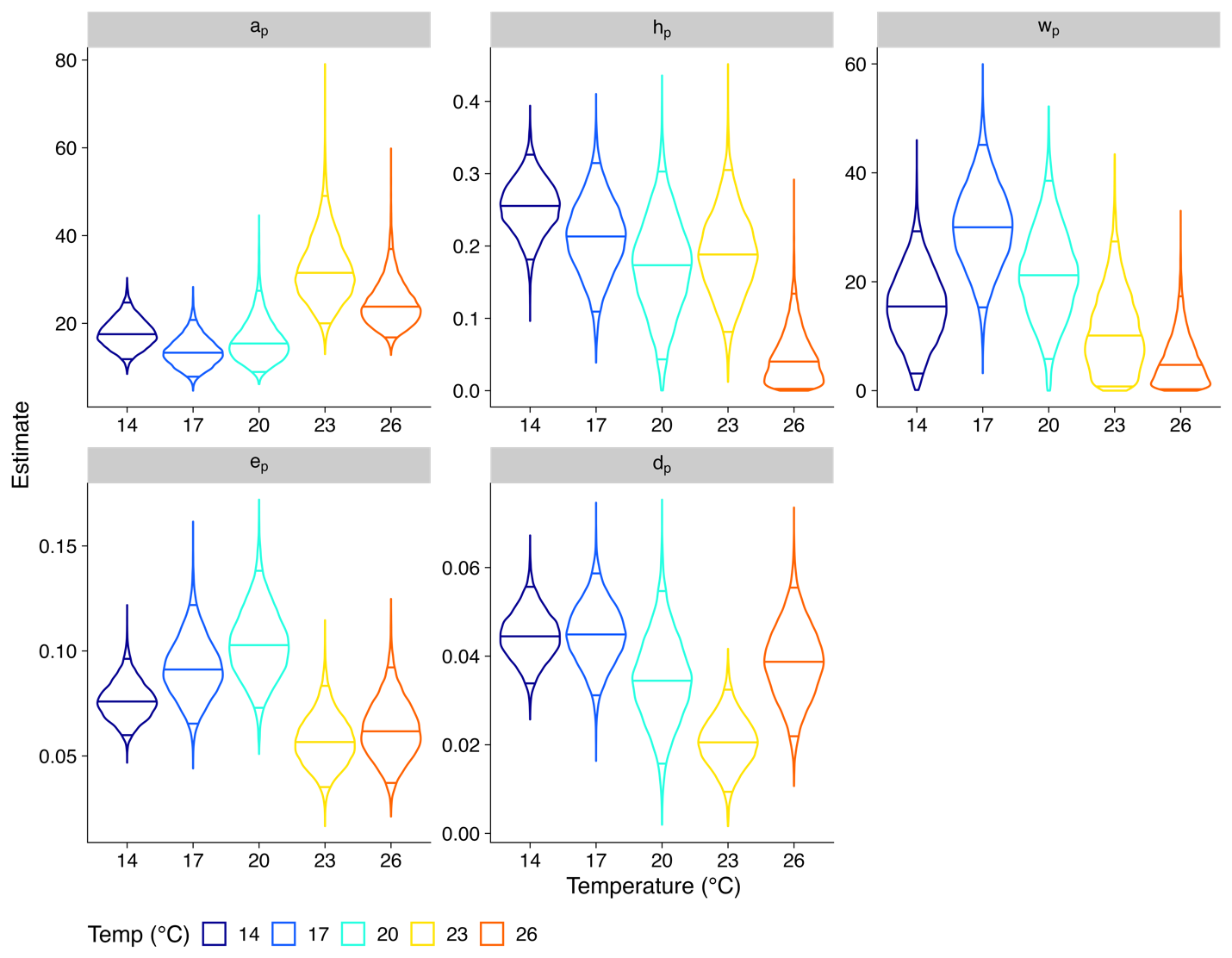
**

Figure C3: **Posterior distribution of parameter estimates related to hydra dynamics from three-species fits.** Horizontal lines in violin plots show 2.5%, 50%, and 97.5% quantiles.

*
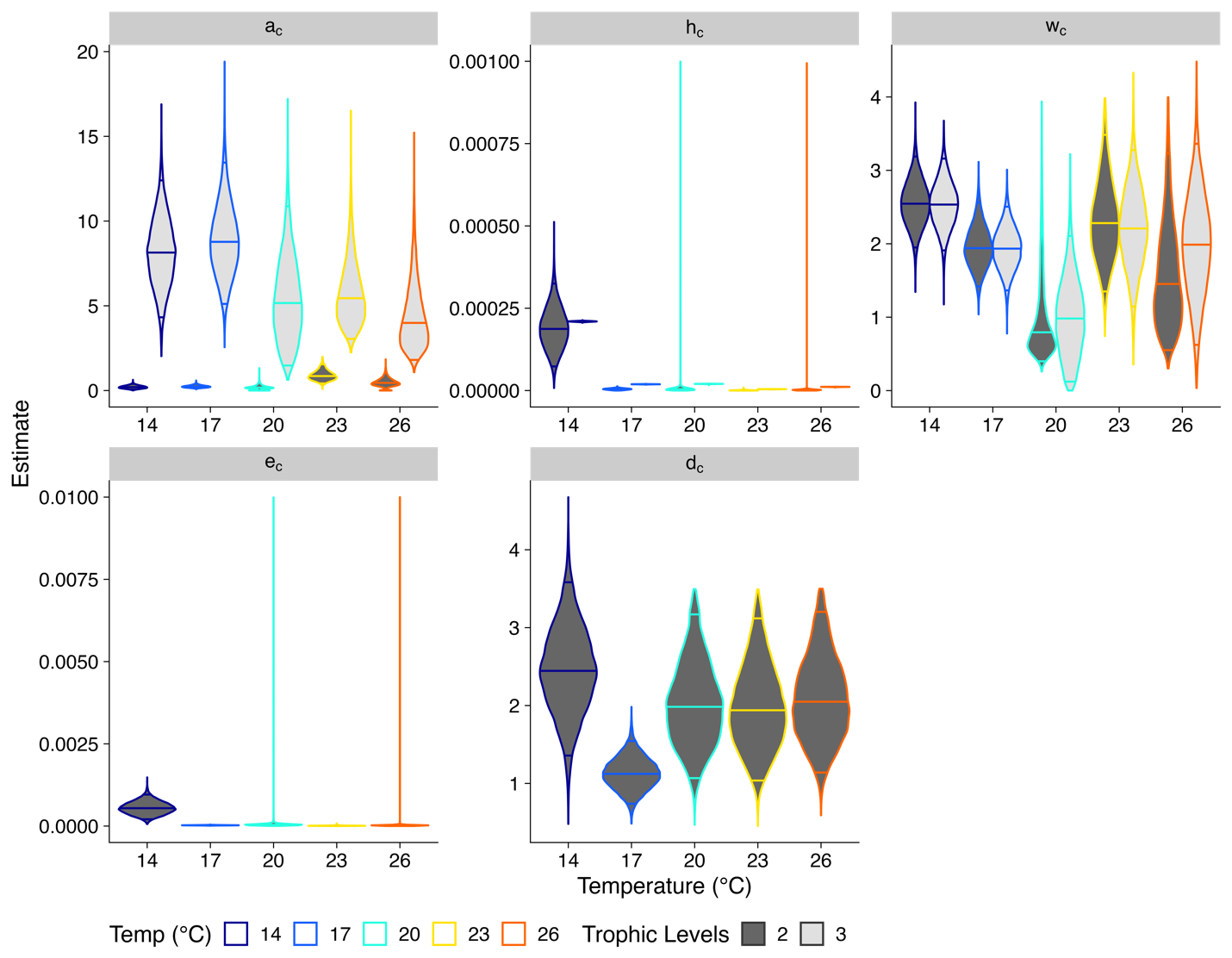
*

Figure C4: **Posterior distribution of parameter estimates related to daphnia dynamics.** Horizontal lines in violin plots show 2.5%, 50%, and 97.5% quantiles. Darker filled violin plots show posterior distributions from treatments without hydra while lighter filled violin plots show posterior distributions from treatments with hydra*.*

**
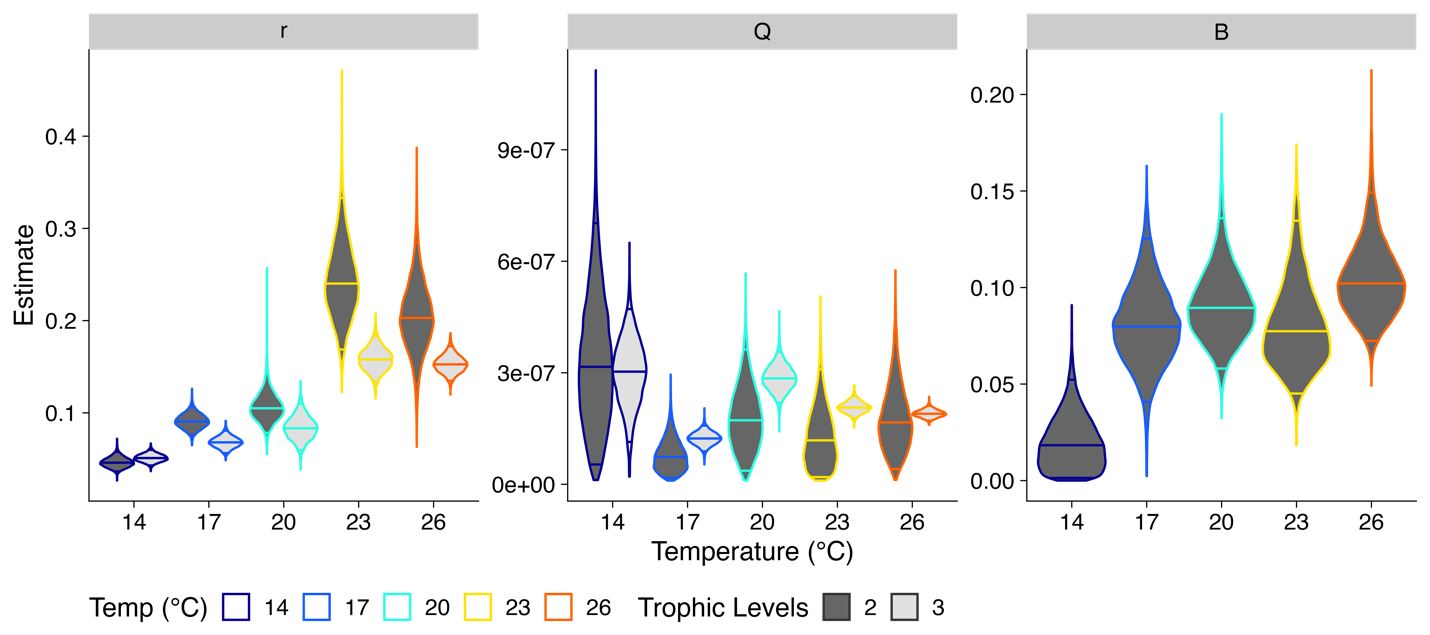
**

Figure C5: **Posterior distribution of parameter estimates related to algae dynamics.** Horizontal lines in violin plots show 2.5%, 50%, and 97.5% quantiles. Darker filled violin plots show posterior distributions from treatments without hydra while lighter filled violin plots show posterior distributions from treatments with hydra*.*
